# Self‐Sustaining Lactate Depletion Nanoplatform Remodels Tumor Microenvironment and Augments Synergistic Photodynamic/Photothermal/Chemodynamic/Starvation Therapy for Eradication of Colon Cancer

**DOI:** 10.1002/advs.76367

**Published:** 2026-06-26

**Authors:** Yu‐E. Wang, Shibo Zhao, Wenying Wu, Zhiwei Duan, Yuan Li, Xiangyu Zeng, Liang Hong, Yan Chen, Ling Tao, Kewu Zeng, Chaoda Xiao, Xiangchun Shen

**Affiliations:** ^1^ State Key Laboratory of Discovery and Utilization of Functional Components in Traditional Chinese Medicine & School of Pharmaceutical Sciences Guizhou International Science & Technology Cooperation Base for Druggability Research of Natural Medicines The High Efficacy Application of Natural Medicinal Resources Engineering Center of Guizhou Province the Key Laboratory of Optimal Utilization of Natural Medicine Resources Guizhou Medical University Guizhou China; ^2^ School of Biology and Engineering Guizhou Medical University Guizhou China; ^3^ Key Laboratory of Novel Anti‐Cancer Drug Targets Discovery and Application School of Pharmaceutical Sciences Guizhou Medical University Guizhou China; ^4^ National Key Laboratory of Natural and Biomimetic Drugs Peking University Beijing China

**Keywords:** colon cancer, lactate depletion, synergistic therapy, tumor microenvironment

## Abstract

Colon cancer remains a formidable therapeutic challenge due to its high malignancy and limited treatment options. To overcome this, we develop a multifunctional nanoplatform (HILA) using hollow mesoporous manganese dioxide (HMnO_2_) to co‐load lactate oxidase (LOX) and the photosensitizer indocyanine green (ICG), coated with hyaluronic acid (HA) for targeted delivery to CD44‐overexpressing tumor cells. Within the tumor microenvironment (TME), LOX converts lactate to more acidic pyruvate and H_2_O_2_, which subsequently react with HMnO_2_ to produce O_2_, establishing a self‐sustaining HMnO_2_‐LOX catalytic cycle that continuously depletes lactate (inducing tumor starvation) while alleviating hypoxia. The acidic pyruvate accelerates HMnO_2_ degradation, releasing ICG and Mn^2+^ ions. Under near‐infrared irradiation, the generated O_2_ facilitates photodynamic/photothermal therapy (PDT/PTT) of ICG, while Mn^2+^ catalyzes H_2_O_2_ to generate hydroxyl radicals (•OH) for enhanced chemodynamic therapy (CDT). Crucially, the coordinated TME remodeling, (featuring lactate exhaustion, pH reduction, and O_2_/H_2_O_2_ accumulation) creates a positive feedback loop that fuels PDT/PTT/CDT/starvation therapy. This self‐reinforcing cycle integrates PDT/PTT/CDT/starvation therapy, demonstrating potent tumor suppression in vivo and offering a paradigm for metabolic modulation‐synergized oncotherapy.

## Graphical abstract

The HILA nanoplatform integrates HMnO_2_, LOX, and ICG to orchestrate multimodal therapy (starvation, chemodynamic, photodynamic, and photothermal) for achieving efficient colon cancer eradication. The established HMnO_2_‐LOX catalytic cycle continuously consumes lactate, acidifys microenvironment, relieves hypoxia, and generats reactive oxygen species (ROS), creating a positive feedback loop for accelerating HILA degradation and amplifing photodynamic, and photothermal synergestic therapy.

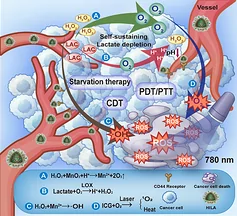

## 1 Introduction

Colon cancer, a prevalent malignancy of the digestive system, presents significant clinical challenges due to its high propensity for metastasis and the development of therapeutic resistance [1]. While surgical intervention, radiotherapy, and chemotherapy remain the cornerstone of conventional treatment modalities, each approach carries substantial limitations. Surgical resection often leads to substantial trauma and postoperative complications; radiotherapy lacks tissue specificity, resulting in collateral damage to surrounding healthy tissues; and chemotherapeutic agents cause systemic toxicity manifesting as bone marrow suppression and gastrointestinal disturbances [2]. These limitations underscore the urgent need for developing novel therapeutic strategies with enhanced efficacy and reduced systemic toxicity.

The tumor microenvironment (TME) not only provides essential support for tumor cell proliferation, invasion, and distant metastasis but also promotes therapeutic resistance through multiple mechanisms, such as enhancing drug efflux capacity and impairing drug penetration into tissues, thereby significantly limiting the efficacy of both chemotherapy and targeted agents [3]. Recent advances in TME research have revealed distinctive physiological characteristics that differentiate malignant tissues from their normal counterparts, including acidic pH, hypoxic conditions, glutathione (GSH) overexpression, and elevated hydrogen peroxide (H_2_O_2_) levels [4]. A central feature of tumor metabolic reprogramming is the Warburg effect, wherein tumor cells preferentially undergo glycolysis even under aerobic conditions, resulting in substantial lactate production [5]. The accumulation of lactate further acidifies the TME and activates the HIF‐1α/VEGF signaling pathway, which promotes tumor angiogenesis [6] and facilitates the infiltration of immunosuppressive cells, such as M2‐type macrophages and regulatory T cells, collectively accelerating malignant progression [7]. Recent studies have also revealed that lactate acts as a precursor molecule inducing a novel epigenetic modification known as protein lactylation, which directly regulates the activity of key proteins such as p53 and MRE11, thereby influencing tumor suppressor functions and DNA damage repair processes, and ultimately promoting tumorigenesis and chemoresistance [8, 9]. Consequently, targeting lactate metabolism and remodeling the TME have emerged as crucial strategies for reversing therapeutic resistance and enhancing treatment response.

Lactate oxidase (LOX) has been demonstrated to catalyze the conversion of lactate into H_2_O_2_ and pyruvate, which is more acidic than lactate, thereby depleting lactate and restoring the immunosuppressive state, providing novel insights for reshaping the tumor immune microenvironment and inhibiting tumor metastasis [10, 11]. However, the lactate‐depleting process of LOX is intrinsically oxygen‐dependent, limiting its sustained efficacy in hypoxic TME. Manganese dioxide (MnO_2_)‐based nanomaterials can catalyze the generation of oxygen from endogenous H_2_O_2_, thereby alleviating tumor hypoxia [12, 13, 14]. Integrating LOX with MnO_2_ presents a promising strategy to construct a self‐sustaining oxygen‐generating cascade catalytic system. This system enables continuous lactate depletion and effective TME remodeling. Specifically, hollow mesoporous MnO_2_ (HMnO_2_) exhibits a high specific surface area and large pore volume, facilitating the efficient loading of diverse therapeutic agents (e.g., small molecules, proteins, genes). Crucially, HMnO_2_ demonstrates enhanced degradation under the acidic and GSH‐rich conditions characteristic of the TME. This property allows for controlled drug release while simultaneously depleting elevated intratumoral GSH levels [15, 16]. Therefore, leveraging HMnO_2_ as a carrier for LOX establishes a self‐amplifying catalytic cycle: i. LOX consumes lactate and O_2_, generating H_2_O_2_ and pyruvate. ii. The produced H_2_O_2_ reacts with HMnO_2_, generating O_2_. iii. This newly generated O_2_ further fuels the LOX‐catalyzed reaction. This positive feedback loop drives continuous lactate consumption, O_2_ and H_2_O_2_ elevation, and acidification (pH reduction), collectively inhibiting tumor energy supply via starvation therapy. Additionally, Mn^2+^ ions released during HMnO_2_ degradation in acid and GSH‐rich TME can catalyze a Fenton‐like reaction with H_2_O_2_, producing highly cytotoxic hydroxyl radicals (•OH) for chemodynamic therapy (CDT) [17, 18]. This CDT synergistically amplifies the antitumor efficacy of the LOX‐mediated starvation therapy.

The highly heterogeneous nature of the TME renders single‐modality therapies inadequate for achieving radical cure in colon cancer. Consequently, the development of intelligent nanoplatforms capable of actively modulating the TME and synergistically integrating multiple treatment modalities has emerged as a pivotal research focus. Photodynamic therapy (PDT) and photothermal therapy (PTT) have garnered significant attention due to their favorable spatiotemporal controllability and low invasiveness [19, 20, 21]. However, the efficacy of both PDT and PTT is critically dependent on the precise delivery of photosensitizers and the availability of sufficient oxygen. Therefore, their therapeutic outcomes are severely limited by the hypoxic state of the TME and the non‐specific distribution of photosensitizers. Indocyanine green (ICG), an FDA‐approved near‐infrared (NIR) dye, is widely utilized for fluorescence navigation during colorectal surgery [22]. Critically, ICG can simultaneously mediate both PDT and PTT [23, 24]. Given its superior NIR deep‐tissue penetration [25], well‐established biosafety [26], and multimodal synergistic capabilities, ICG‐based phototherapy holds significant promise for clinical translation. Nevertheless, its inherent limitations—including poor stability and inadequate tumor‐targeting capability—significantly hinder its clinical translation [27]. Thus, targeted delivery of ICG to tumor tissues combined with alleviation of tumor oxygen deprivation represents a key strategy for enhancing the efficacy of combined PDT/PTT. The high surface area of HMnO_2_ enables the co‐loading of both ICG and LOX. The O_2_ generated by the HMnO_2_–LOX cascade reaction significantly enhances the efficacy of ICG‐mediated PDT and PTT. This integration effectively combines TME modulation (via lactate depletion and hypoxia alleviation) with multi‐modal PDT/PTT, establishing a self‐reinforcing cascade for amplified antitumor effects, ultimately improving colon cancer therapy.

Based on the aforementioned rationale, we designed a multifunctional nanosystem (designated HILA) employing HMnO_2_ as the core carrier. The HILA platform simultaneously encapsulates ICG and lactate oxidase LOX, with surface modification via hyaluronic acid (HA), as Scheme 1 shown. This HA coating enables CD44 receptor‐mediated active tumor targeting [28], while concurrently enhancing nanoparticle stability, biocompatibility, and systemic circulation longevity [29]. The therapeutic action of HILA operates through a self‐reinforcing therapeutic cascade: The HMnO_2_–LOX catalytic cycle continuously converts lactate to pyruvate with H_2_O_2_ generation, while HMnO_2_ decomposes endogenous/exogenous H_2_O_2_ to produce O_2_. This process simultaneously alleviates tumor hypoxia (critical for enhancing ICG‐based PDT/PDT), sustainably depletes lactate (starvation therapy), and lowers pH (accelerating the degradation of HMnO_2_ and activating ICG and Mn^2+^ release). The synergistic quadruple therapy of starvation therapy by LOX‐driven lactate depletion, CDT mediated by Mn^2+^ catalyzing a Fenton‐like reaction, and PDT/PTT was also ingeniously integrated into this process, establishing a self‐amplifying feedback. Collectively, we have developed a novel paradigm that remodels the tumor microenvironment and augments synergistic multimodal therapy, providing new ideas and experimental basis for the treatment of colon cancer.

**SCHEME 1:**
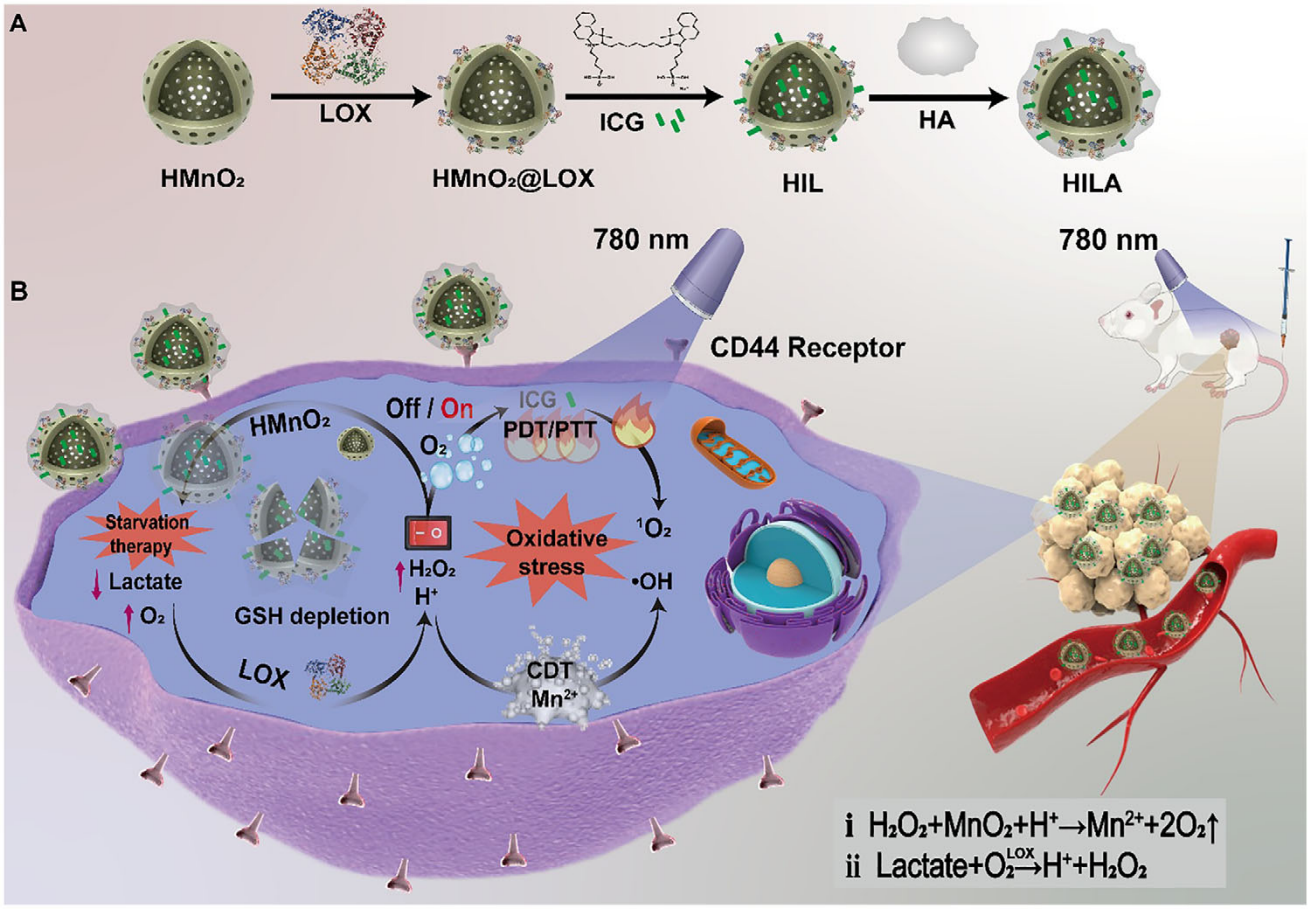
(A) Preparation process of multifunctional HILA nanoparticles and (B) significantly amplified antitumor effects via synergistic action of ROS‐GSH/pH/O_2_ axis. The structural representation of LOX was retrieved from the RCSB Protein Data Bank (PDB ID: 2J6X).

## 2 Results and Discussion

### 2.1 Preparation and Characterization of HILA

The stepwise fabrication of the HILA nanoplatform is illustrated in Figure 1A. Pristine HMnO_2_ was aminated to covalently immobilize LOX via amide bonds, forming HMnO_2_@LOX. Subsequently, ICG was loaded into the hollow mesoporous structure of HMnO_2_@LOX to form the ICG‐loaded nanoparticle (HIL), followed by surface adsorption of HA to produce the final HILA construct. The visual evolution of intermediate products is shown in Figure S1.

**FIGURE 1:**
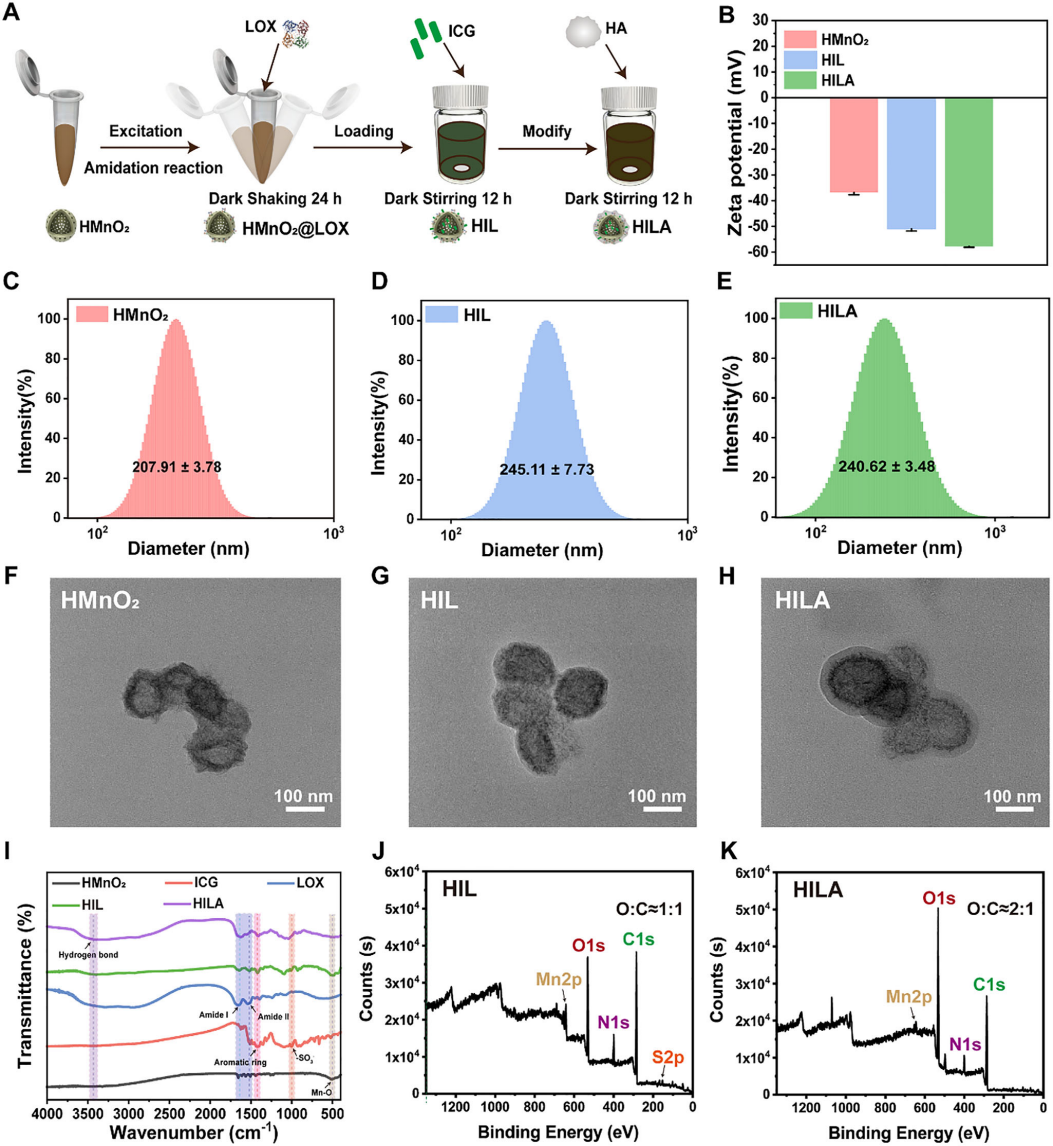
Preparation and characterization of HILA. (A) Schematic of HILA preparation. (B) Zeta potential of HMnO_2_, HIL, and HILA. (C–E) Particle size distribution curves of HMnO_2_ (C), HIL (D), and HILA (E). (F–H) TEM images of HMnO_2_ (F), HIL(G), and HILA(H). (I) FT‐IR spectra of HMnO_2_, ICG, LOX, HIL, and HILA. (J,K) XPS Spectrum of HIL (J) and HILA (K). Data are presented as mean ± SD, *n* = 3.

The physicochemical properties were systematically characterized to monitor the structural evolution during each modification step. The surface zeta potential progressively decreased from −36.76 mV (pristine HMnO_2_) to −57.79 mV (HILA) during functionalization (Figure 1B). This negative shift stems from the cumulative contribution of ionized anionic moieties from ICG (sulfonate groups), LOX (isoelectric point at 4.3), and the HA coating (deprotonated carboxyl groups) measured in deionized water. Dynamic light scattering (DLS) analysis revealed that the hydrodynamic diameter increased from 207.91 ± 3.78 nm (HMnO_2_) to 245.11 ± 7.37 nm for the intermediate HIL (Figure 1C,D), reflecting the expansion of the framework due to LOX immobilization and ICG loading. Upon HA coating, the final HILA formulation exhibited a slight size reduction to 240.62 ± 3.48 nm (Figure 1E), suggesting that the HA coating contributes to surface stabilization and a more uniform size distribution. Transmission electron microscopy (TEM) imaging provided morphological evidence supporting this structural evolution. HMnO_2_ exhibited a characteristic hollow mesoporous structure characterized by a distinct gray core and dark edges (Figure 1F and Figure S2) and the mesopores predominantly distributed in the range of 1.0–3.0 nm (Figure S3). In HIL (Figure 1G), the central region appeared significantly darker with thickened edges, indicating successful loading of the payload. For HILA (Figure 1H), a thin, film‐like layer was clearly observed on the particle surface, confirming uniform HA coating. Notably, the hydrodynamic diameters measured by DLS were significantly larger than the core sizes observed in TEM (∼100–150 nm). This discrepancy is primarily attributed to the distinct measurement principles of the two techniques [30].

Multimodal spectroscopic characterization provided compelling evidence for the successful assembly and molecular‐level integrity of the HILA nanoplatform. Characteristic absorption peaks (710 nm and 780 nm) and fluorescence emission (780–840 nm) of ICG were observed in both HIL and HILA (Figure S4), indicating successful ICG loading. Fourier transform infrared spectroscopy (FT‐IR) analysis delivered crucial molecular‐level evidence confirming the successful formation of the nanoparticles (Figure 1I and Figure S5). The spectra of HIL and HILA displayed prominent amide bond vibrations at 1650 and 1550 cm^−1^ (originating from LOX), characteristic ICG peaks at 900–1100 and 1400–1500 cm^−1^, and Mn─O vibrations at 490 cm^−1^, confirming the effective encapsulation of LOX and ICG within HMnO_2_. Furthermore, X‐ray photoelectron spectroscopy (XPS) revealed changes in surface composition. Compared to HMnO_2_, the appearance of C, N, and S elements in HIL nanoparticles is attributed to the successful loading of LOX and ICG (Figure 1J). And the high‐resolution XPS scans of the S 2p region in HIL revealed the presence of C─S and O═S bonds characteristic of sulfonate groups in ICG, further demonstrating the successful loading of ICG (Figure S6). Following HA modification, HILA exhibited increased O content (C:O atomic ratio shifted from 1:1 to 1:2) and significantly attenuated signals of Mn, N, and S (Figure 1K), indicating effective surface coverage by the HA layer. These results collectively confirm the successful loading of LOX and ICG, as well as the successful surface modification of HA.

Furthermore, UV–Vis spectrophotometric analysis demonstrated that the HILA nanoplatform achieved high‐efficiency ICG encapsulation, with a drug loading (DL) capacity of 14.11 ± 0.23% and encapsulation efficiency (EE) of 84.10 ± 1.34% (Table S1). Successful immobilization of LOX was further validated by parallel quantitative BCA assays, which revealed a DL of 16.40 ± 0.93% and an EE of 81.45 ± 4.61% (Table S2). These results collectively demonstrate that HMnO_2_‐based nanoparticles possess excellent drug loading capacity, rendering them suitable for the development of multifunctional nanocomposite systems. Additionally, stability tests revealed that HILA maintained consistent particle size without significant changes over a 7‐day incubation period in simulated physiological media, including 10% FBS, PBS, and DMEM (Figure S7), demonstrating excellent colloidal stability and adaptability to biological environments. Collectively, the physicochemical characterization of these systems provides conclusive evidence for the successful construction of HILA and its intermediate complexes.

### 2.2 In Vitro Catalytic Performance of HILA

This study demonstrates the superior catalytic efficiency of the HILA in vitro. Upon entering the TME, the loaded LOX catalyzes the conversion of lactate into H_2_O_2_ and pyruvate in the presence of O_2_. Concurrently, the carrier material HMnO_2_ undergoes a cascade reaction with the in situ‐generated H_2_O_2_ to continuously generate O_2_, which sustains the catalytic cycle and promotes lactate depletion (Figure 2A). This self‐sustaining cyclic process not only depletes lactate, thereby disrupting substrate availability for tumor energy metabolism and inhibiting tumor growth through metabolic starvation, but also modulates the levels of O_2_ and H_2_O_2_ within the TME. The production of pyruvate contributes to an increasingly acidic environment, which, in coordination with GSH, accelerates the degradation of HMnO_2_ and facilitates the release of the photosensitizer ICG and Mn^2+^ ions. The released Mn^2+^ ions subsequently participate in a Fenton‐like reaction with intracellular H_2_O_2_ derived from lactate metabolism, generating cytotoxic •OH to exert a CDT effect. Upon NIR irradiation, the released ICG simultaneously mediates PDT through the production of singlet oxygen (^1^O_2_) and PTT via localized hyperthermia. Critically, the O_2_ generated from the catalytic cycle alleviates tumor hypoxia, thereby substantially potentiating the efficacy of both PDT and PTT.

**FIGURE 2:**
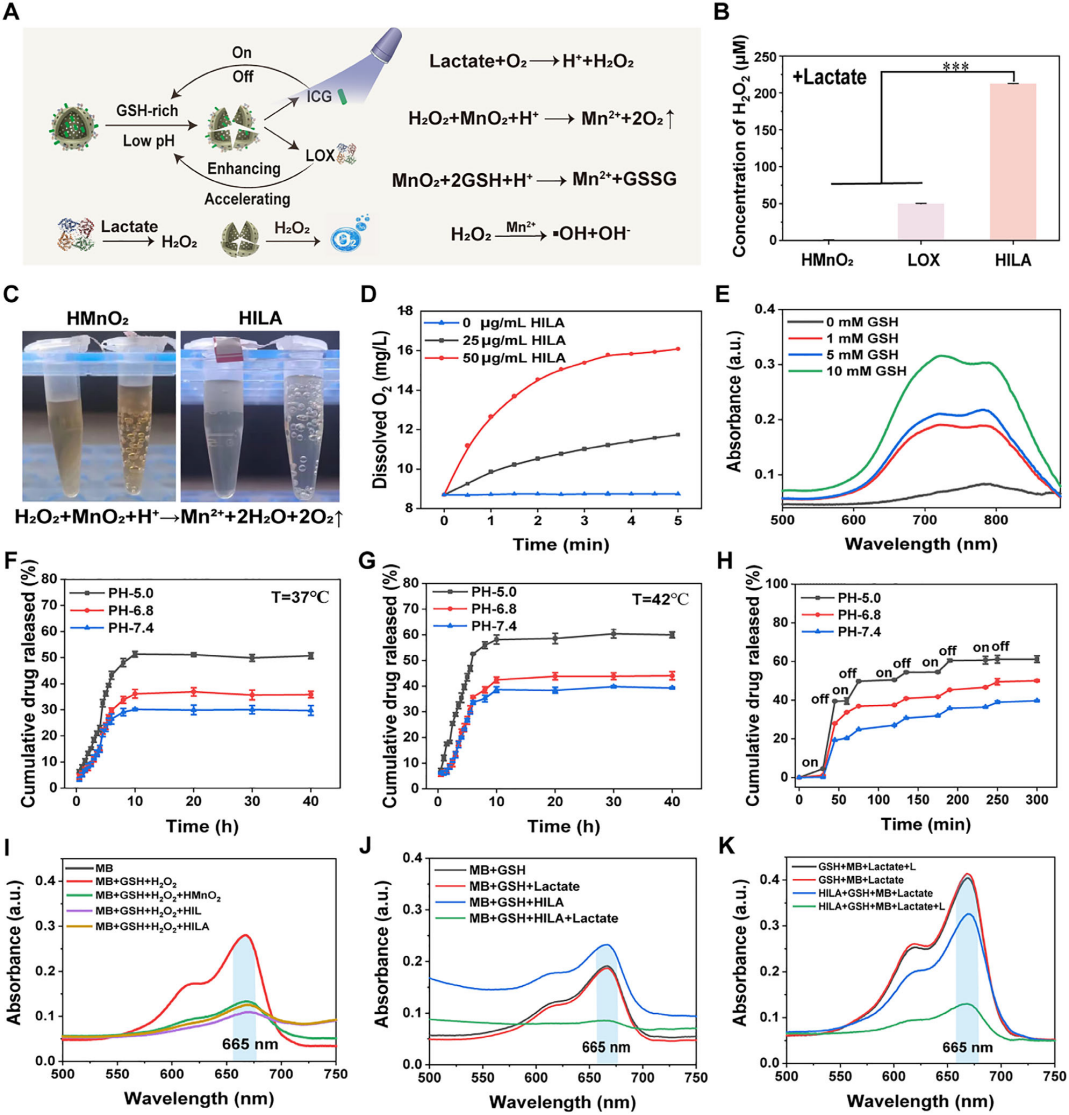
In Vitro Catalytic Performance of HILA. (A) Schematic diagram of the cascade catalytic reaction mechanism, highlighting the generation of H_2_O_2_, O_2_, and •OH. (B) Quantification of H_2_O_2_ generation by different formulations in the presence of lactate (5 mg/mL). (C) Representative photographs showing oxygen bubble generation during the reaction between HILA and H_2_O_2_. (D) Oxygen evolution curves of HILA at various concentrations reacting with H_2_O_2_. (E) UV absorption spectra indicating ICG release from HILA in solutions with varying GSH concentrations. (F,G) ICG release profiles from HILA in PBS buffers of different pH values at 37°C (F) and 42°C (G). (H) Cumulative ICG release profiles from HILA under NIR irradiation in PBS buffers of different pH values. (I) MB absorption spectra after H_2_O_2_ reaction with different nanoparticles (HMnO_2_, HIL, and HILA) under pH 5.5 and 10 mM GSH conditions. (J) MB absorption spectra of HILA in the presence of lactate (40 mM). (K) MB absorption spectra of HILA under NIR irradiation in a simulated TME containing lactate (40 mM). Data are presented as mean ± SD, *n* = 3. Statistic significances between every two groups was calculated by *two‐tailed* *Student's t‐test*. ^***^ *p* < 0.001.

#### 2.2.1 The HMnO_2_–LOX Catalytic Cycle Resulting in Lactate Depletion and H_2_O_2_/O_2_ Generation

HILA leverages a synergistic interaction between LOX and HMnO_2_ to establish a self‐sustaining catalytic cycle. This catalytic efficiency was validated experimentally. As shown in Figure 2B, LOX catalyzes the conversion of lactate to H_2_O_2_, generating a concentration of 50.58 µM. The HILA group produces 4.25‐fold more H_2_O_2_ than LOX alone, attributable to the presence of HMnO_2_ within the nanoparticles, which converts the generated H_2_O_2_ into O_2_, thereby establishing a catalytic cycle that enables the continuous oxidation of lactate and sustained H_2_O_2_/O_2_ production. Furthermore, HILA exhibited stable and highly efficient catalytic activity across a lactate concentration gradient ranging from 2 to 20 mg/mL (Figure S8). The O_2_ generation capacity of the HILA system was also characterized (Figure 2C,D). Distinct oxygen bubbles were observed in the HMnO_2_ and HILA groups upon H_2_O_2_ addition (Figure 2C), with production rates increasing in a concentration‐ and time‐dependent manner (Figure 2D). Further comparisons were made between free LOX and HILA regarding O_2_ consumption during lactate catalysis (Figure S9). Upon lactate addition, free LOX rapidly consumed dissolved oxygen, whereas HILA exhibited a much more gradual decline. This indicates that the in situ O_2_ regeneration by HMnO_2_ effectively compensates for the oxygen consumption by LOX. These findings confirm that HILA preserves the biocatalytic function of LOX while establishing a self‐amplifying therapeutic cycle: LOX catalyzes the conversion of lactate into H_2_O_2_ and pyruvate, while HMnO_2_ subsequently decomposes the generated H_2_O_2_ into O_2_, thereby replenishing the substrate required for LOX activity. This spatiotemporally coupled mechanism simultaneously alleviates tumor hypoxia and drives continuous lactate depletion, positioning HILA as a robust platform for self‐sustaining tumor metabolic intervention.

#### 2.2.2 Degradation Property of HILA

The degradation behavior of HILA under acidic conditions and in the presence of GSH was systematically investigated, using ICG release as an indicator. As shown in Figure 2E, ICG release increased with rising GSH concentration, primarily due to GSH‐triggered degradation of HMnO_2_ carrier [31, 32]. The release profile also exhibited distinct pH‐dependency. At 37°C (Figure 2F), the cumulative ICG release at pH 5.0 over 40 h was significantly higher than that at pH 6.8 and 7.4, confirming acid‐triggered degradation. Additionally, the presence of H_2_O_2_ enhanced ICG release (Figure S10), mainly because the reaction between HMnO_2_ and H_2_O_2_ generates Mn^2+^ and O_2_, further disrupting the nanostructure. Furthermore, a thermo‐responsive release feature was identified. Compared to 37°C, ICG release at various pH levels was significantly accelerated at 42°C (Figure 2G), likely due to enhanced molecular diffusion and structural loosening [33]. To further validate this phenomenon, NIR irradiation was applied to activate the PTT of ICG, thereby triggering and monitoring ICG release in situ. The results revealed a sharp increase in ICG release upon laser irradiation, confirming the promoting effect of temperature on ICG liberation (Figure 2H). Collectively, these results demonstrate that HILA responds to multiple TME stimuli (acidity, GSH, H_2_O_2_) and external NIR irradiation. Specifically, the HMnO_2_‐LOX catalytic cycle synergistically promotes carrier degradation by generating a localized acidic environment and H_2_O_2_, while the PTT effect facilitates on‐demand drug release, thereby enhancing tumor‐specific therapeutic efficacy and biosafety.

#### 2.2.3 Catalytic Generation of •OH

The ability of HILA to convert H_2_O_2_ into highly cytotoxic •OH was evaluated by monitoring the characteristic absorption decay of methylene blue (MB) at 665 nm. Under simulated tumor microenvironment conditions (pH 5.0, GSH 10 mM), HMnO_2_, HIL, and HILA all demonstrated comparable abilities to react with H_2_O_2_ and generate •OH, as evidenced by a significant decrease in the characteristic MB absorption peak (Figure 2I). This suggests that •OH generation is predominantly driven by TME‐triggered Mn^2+^ release. Furthermore, the production of •OH by HILA increases progressively with prolonged reaction time (Figure S11), indicating sustained •OH generation under HMnO_2_ catalysis. To further elucidate the mechanism of •OH generation through the interaction between HILA and H_2_O_2_, the catalytic production of •OH by HILA was systematically evaluated under varying GSH concentrations and pH conditions. The results demonstrated that the yield of •OH increased with rising GSH levels (Figure S12A), and significantly higher •OH generation was observed under mildly acidic conditions (pH 5.0, Figure S12B). These findings indicate that HILA undergoes degradation in a mildly acidic or GSH‐rich environment, leading to the release of Mn^2+^, which subsequently participates in a Fenton‐like reaction with H_2_O_2_ to produce •OH. Notably, the introduction of lactate (2.5 mg/mL) effectively triggered •OH production (Figure 2J), attributed to the HMnO_2_‐LOX catalytic cycle that converts lactate into H_2_O_2_ while concurrently establishing a more acidic microenvironment. This acidic shift promotes the disintegration of the HMnO_2_ framework, facilitating Mn^2+^ release and enabling its reaction with H_2_O_2_ to generate •OH, thereby exerting CDT effects. Furthermore, studies revealed that under laser irradiation in a lactate‐rich environment (40 mM), HILA generated a greater amount of •OH (Figure 2K). To investigate whether temperature influences the production of •OH (Figure S13), it was found that elevated temperature led to increased •OH generation. This enhancement is attributed to the heat‐accelerated HILA degradation and Mn^2+^ release, which enhance the Fenton‐like reaction kinetics. Collectively, these results suggest that HILA initiates a self‐sustained cascade catalysis within the TME, continuously consuming lactate and remodeling the microenvironment to induce a robust ROS storm for synergistic cancer therapeutics.

### 2.3 Evaluation of HILA's Photodynamic Effects and Photothermal Capabilities

The ICG released from HILA absorbs NIR light and converts surrounding O_2_ into ^1^O_2_, thereby killing tumor cells through PDT (Figure 3A). Simultaneously, it can convert light energy into heat energy, serving as a photothermal agent for PTT [34].

**FIGURE 3:**
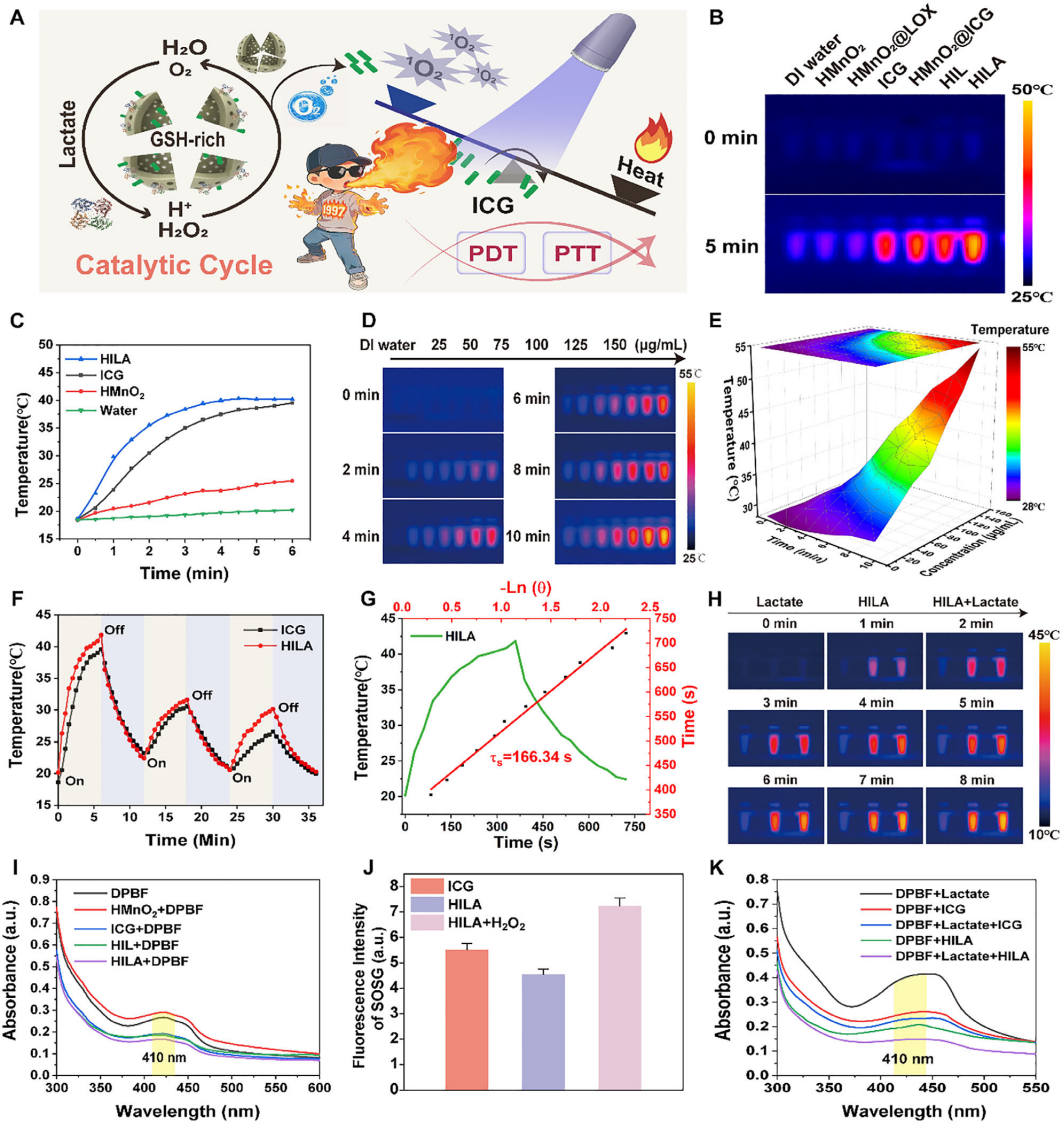
Evaluation of HILA's Photodynamic Effects and Photothermal Capabilities. (A) Schematic diagram of enhanced PDT/PTT mechanism based on the HMnO_2_‐LOX cascade reaction. The anime character in the scheme was produced with the assistance of Dou Bao AI. (B) Infrared thermal images of HILA and its component nanoparticles under NIR irradiation. (C) Corresponding temperature elevation curves of the groups in (B). (D) Infrared thermal images of HILA at varying concentrations. (E) Heatmap of HILA temperature varying with time and concentration. (F) Photothermal stability comparison between HILA (equivalent ICG concentration: 50 µg/mL) and free ICG over three laser on‐off cycles. (G) Linear fitting relationship between the cooling time and ‐lnθ used for calculating photothermal conversion efficiency. (H) Infrared thermal images of HILA in a lactate‐enriched environment (40 mM) simulating the TME. (I) UV–Vis absorption spectra of DPBF probes in aqueous solutions containing different formulations after NIR irradiation. (J) Effect of exogenous H_2_O_2_ on ^1^O_2_ generation by HILA. (K) Effect of lactate (40 mM) on ^1^O_2_ generation by HILA. Data are presented as mean ± SD, *n* = 3.

#### 2.3.1 Photothermal Effect of HILA

The photothermal performance of HILA was systematically evaluated under 780 nm laser irradiation (1 W/cm^2^). Infrared thermal images (Figure 3B) and temperature curves (Figure 3C) revealed that ICG‐containing formulations exhibited a significant photothermal effect. Notably, HILA achieved the highest temperature increase (ΔT = 22.80°C), surpassing free ICG (ΔT = 20.38°C). This effect showed a positive correlation with both concentration and irradiation duration (Figure 3D,E, and Figure S14). Furthermore, photostability assessment demonstrated that HILA maintained favorable stability over three heating‐cooling cycles, with a peak temperature decrease of only 9.65°C compared to 14.85°C for free ICG (Figure 3F). This indicates that HILA nanoparticles improved the photostability of ICG. The photothermal conversion efficiency (η) of HILA was analyzed according to three irradiation‐cooling cycles obtained at a concentration of 100 µg/mL. The linear relationship between ‐lnθ and time was fitted and displayed in Figure 3G, yielding a time constant τ_s_ = 166.34 s and an absorbance A_λ_ = 0.1602. Using Equations S5–S8 provided in the Supporting Information, the η of HILA was calculated to be 33.83%, highlighting its satisfactory photothermal conversion capability.

Furthermore, the photothermal property of HILA was benchmarked against literature‐reported agents (Table S3). The η of HILA (33.83%) is comparable to unmodified black phosphorus (BP, 22.6%–28.4%) and PEGylated BP (∼28.7%) [35], although certain modified BP can reach 64.2% [36]. Gold nanomaterials show shape‐dependent η values (e.g., star‐shaped colloids show efficiencies up to 36% higher than spheres) and long‐term toxicity [37, 38, 39]. While HILA may not surpass all counterparts in photothermal stability, its biodegradability and FDA‐approved components offer distinct clinical translation advantages over BP and gold nanomaterials.

Additionally, under lactate‐enriched conditions (40 mM) simulating the TME, HILA reached a higher terminal temperature (42.25°C) compared to normal medium (38.92°C) (Figure 3H and Figure S15). This improvement is attributed to the HMnO_2_‐LOX self‐sustaining catalytic cycle involving lactate oxidation to H_2_O_2_ followed by its decomposition to O_2_, thereby alleviating hypoxia‐induced self‐quenching of ICG, maintaining its photostability under NIR irradiation.

#### 2.3.2 Photodynamic Effect of HILA

The photodynamic properties of HILA were systematically evaluated by monitoring ^1^O_2_ generation using the 1,3‐Diphenylisobenzofuran (DPBF) decay method or the singlet oxygen fluorescence green (SOSG) fluorescence assay. Upon NIR irradiation (780 nm, 1 W/cm^2^, 5 min), groups containing ICG exhibited a significant reduction in DPBF absorbance (400–450 nm), indicating robust ^1^O_2_ production (Figure 3I). Both HILA and free ICG exhibited a time‐dependent increase in ^1^O_2_ production (Figure S16A,C). Notably, the ^1^O_2_ quantum yield of HILA reached 0.0178, which is approximately 8.9 times higher than that of free ICG (Figure S17). Although ICG generally possesses lower ROS generation efficiency compared to other photosensitizers such as Ce6, BODIPY derivatives, or porphyrins [40], its superior deep‐tissue penetration capabilities [25, 41] and clinical safety [22] make it advantageous. Crucially, the HILA design overcomes ICG's inherent limitations by amplifying PDT efficacy through TME modulation. Specifically, the addition of exogenous H_2_O_2_ (500 µM) enhanced ^1^O_2_ generation in the HILA group (Figure 3J), suggesting that exogenous H_2_O_2_ elevates surrounding oxygen levels by HMnO_2_ catalysis, thereby amplifying PDT efficacy. Furthermore, lactate supplementation (40 mM) markedly increased ^1^O_2_ production in HILA but not in free ICG (Figure 3K), confirming that the HMnO_2_‐LOX catalytic cycle effectively generates oxygen to boost PDT. Collectively, the self‐sustaining lactate depletion system in HILA maintains an adequate O_2_ and H_2_O_2_ supply, synergistically promoting HMnO_2_ decomposition and ICG release, thereby significantly enhancing PDT efficacy.

### 2.4 Tumor Targeting Effect of HILA

#### 2.4.1 Cellular Uptake and Targeting of HILA

Before delving into the antitumor efficacy of HILA at the cellular level, the cellular uptake efficiency, a critical factor determining therapeutic outcomes, was investigated in CT26 mouse colon cancer cells. Leveraging the intrinsic NIR fluorescence of ICG, the strongest fluorescence signal was observed in HILA‐treated cells, significantly surpassing other groups (Figure 4A), indicating enhanced internalization of HA‐modified nanoparticles. To overcome the technical limitations of flow cytometry in the NIR range, coumarin 6 (C6) was employed as an alternative fluorophore probe for quantitative analysis. Flow cytometric results (Figure 4B) corroborated the microscopy findings. To verify the HA‐CD44 receptor‐mediated mechanism, a competitive inhibition assay was performed. Pretreatment with free HA to block CD44 receptors significantly attenuated the fluorescence intensity (Figure 4A), confirming the specificity of the interaction. Furthermore, uptake kinetics studies demonstrated a time‐dependent increase in internalization, peaking at 4 h (Figure 4C,D), which aligns with the time‐dependent cytotoxicity observed subsequently. Collectively, these results demonstrate that HILA is efficiently internalized into CT26 cells via CD44 receptor‐mediated endocytosis [42], providing solid experimental evidence for its application in tumor‐targeted therapy.

**FIGURE 4:**
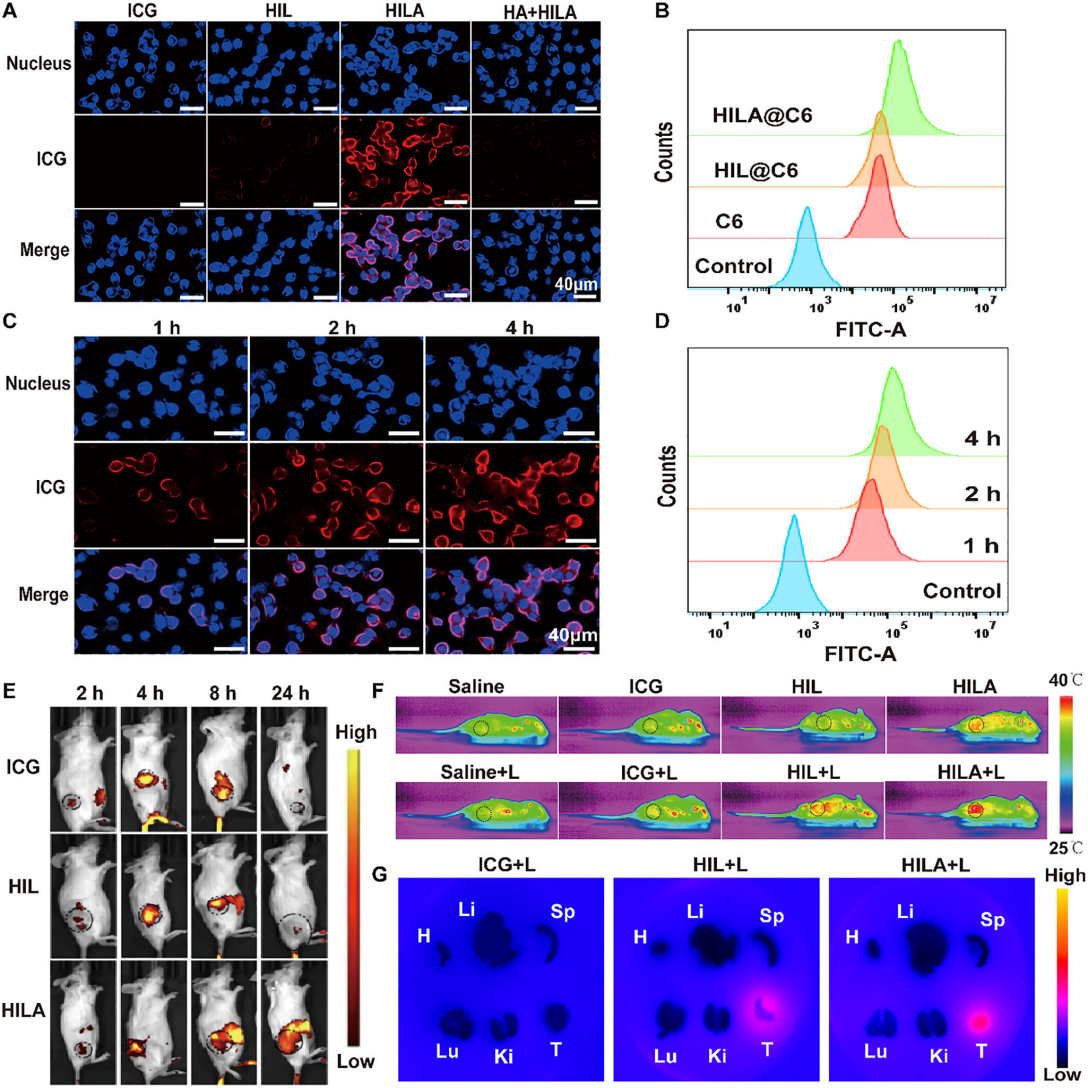
Cellular Uptake and in vivo Tumor Targeting Efficiency of HILA. (A) Fluorescence microscopy images of CT26 cells incubated with different nanoparticles (scale bar = 40 µm). (B) Quantitative analysis of fluorescence intensity corresponding to (A). (C) Fluorescence microscopy images of CT26 cells incubated with HILA for various durations (scale bar = 40 µm). (D) Quantitative analysis of time‐dependent cellular uptake. (E) Time‐lapse in vivo fluorescence images of CT26 tumor‐bearing mice post‐injection. (F) Infrared thermal images of tumor sites under laser irradiation 24 h postinjection. (G) Ex vivo fluorescence images of major organs and tumors harvested 24 h postinjection.

#### 2.4.2 In Vivo Tumor Targeting of HILA

To investigate the in vivo bio‐distribution of HILA, ICG fluorescence imaging was performed on CT26 tumor‐bearing mice, comparing free ICG, HIL, and HILA. As shown in Figure 4E, mice injected with free ICG exhibited weak fluorescence signals in the tumor region that largely disappeared after 24 h, indicating rapid metabolism and poor tumor accumulation capacity. In contrast, the HILA group displayed progressively enhanced fluorescence signals in the tumor region within 8 h postinjection, maintaining high intensity even after 24 h (Figure S18), demonstrating excellent tumor targeting and retention capabilities. Consistent with these findings, infrared thermal imaging (Figure 4F) under laser irradiation (2 W/cm^2^, 5 min) revealed that the HILA group achieved the most potent photothermal response. The temperature at the tumor site in the HILA group increased by approximately 10 °C (Figure S19), with both heating rate and magnitude surpassing those of the ICG and HIL groups. This confirms the enhanced in vivo photothermal conversion efficiency driven by HA‐mediated tumor targeting. Moreover, ex vivo photothermal imaging of major organs 24 h postinjection (Figure 4G) clearly demonstrated specific HILA accumulation in tumor tissue with a distinct photothermal signal, corroborating the in vivo results. Collectively, HA modification substantially prolonged the circulation time and enhanced the tumor‐targeting efficiency of HILA, laying a solid foundation for improved antitumor efficacy.

### 2.5 In Vitro Cytotoxicity

#### 2.5.1 In Vitro Biosafety

The in vitro biosafety of different formulation groups (ICG, HMnO_2_, HMnO_2_@LOX, and HILA) prior to cellular investigation was comprehensively evaluated by the MTT assay on mouse fibroblast L‐929 cells under both dark conditions and NIR irradiation (780 nm, 1 W/cm^2^, 2 min). Under dark conditions (Figure S20A), HMnO_2_@LOX and HMnO_2_ (equivalent to the carrier in HMnO_2_@LOX) exhibited significant cytotoxicity at LOX concentrations exceeding 11.5 µg/mL, whereas HILA maintained high cell viability. This confirms that the HA coating effectively shields the potential toxicity of the core components (LOX and HMnO_2_), ensuring superior biocompatibility. Under NIR irradiation (Figure S20B), free ICG (30 µg/mL) significantly reduced cell viability to 67.76%, whereas HILA maintained a higher viability of 85.28%. This reduced phototoxicity suggests that HA modification limits nonspecific cellular uptake in normal cells, thereby minimizing off‐target effects. Furthermore, HILA showed negligible toxicity in the dark and only mild effects upon irradiation at concentrations up to 20 µg/mL ICG and 23 µg/mL LOX (Figure S20A). These results establish a safe dosage window (ICG concentrations below 20 µg/mL or LOX concentrations below 23 µg/mL) for subsequent experiments, confirming that HILA possesses excellent biocompatibility in non‐targeted tissues.

#### 2.5.2 In Vitro Cytotoxicity

The in vitro antitumor effect of HILA was systematically assessed in CT26 cells via an MTT assay. To optimize incubation conditions, time‐dependent phototoxicity was first assessed (Figure S21). Cell viability in the HIL and HILA groups dropped below 50% after 4 h of incubation, with no significant increase in cytotoxicity upon further extension, identifying 4 h as the optimal duration. Under dark conditions (Figure 5A), high concentrations of HMnO_2_, HMnO_2_@LOX, HIL, and HILA exhibited only mild cytotoxicity. Notably, at an ICG concentration of 17.5 µg/mL (LOX 20.125 µg/mL), the viability of cells treated with HMnO_2_@LOX, HIL, and HILA remained approximately 80%, whereas free ICG showed negligible toxicity (95.99% viability). This confirms that the dark toxicity of these formulations is attributable to the synergistic effect of lactate depletion‐mediated starvation therapy and CDT enabled by the HMnO_2_‐LOX catalytic cycle, rather than the photosensitizer itself. All materials demonstrated inhibition rates below 15% at ICG concentrations ≤17.5 µg/mL, indicating favorable baseline biosafety. Upon NIR irradiation (Figure 5B), HMnO_2_ and HMnO_2_@LOX showed no significant phototoxicity compared to blank controls. In contrast, ICG, HIL, and HILA exhibited concentration‐dependent phototoxicity. HILA achieved the most potent efficacy, reducing cell viability to approximately 20% at an equivalent ICG concentration of 17.5 µg/mL, highlighting the enhanced PDT/PTT effect conferred by the nanostructure.

**FIGURE 5:**
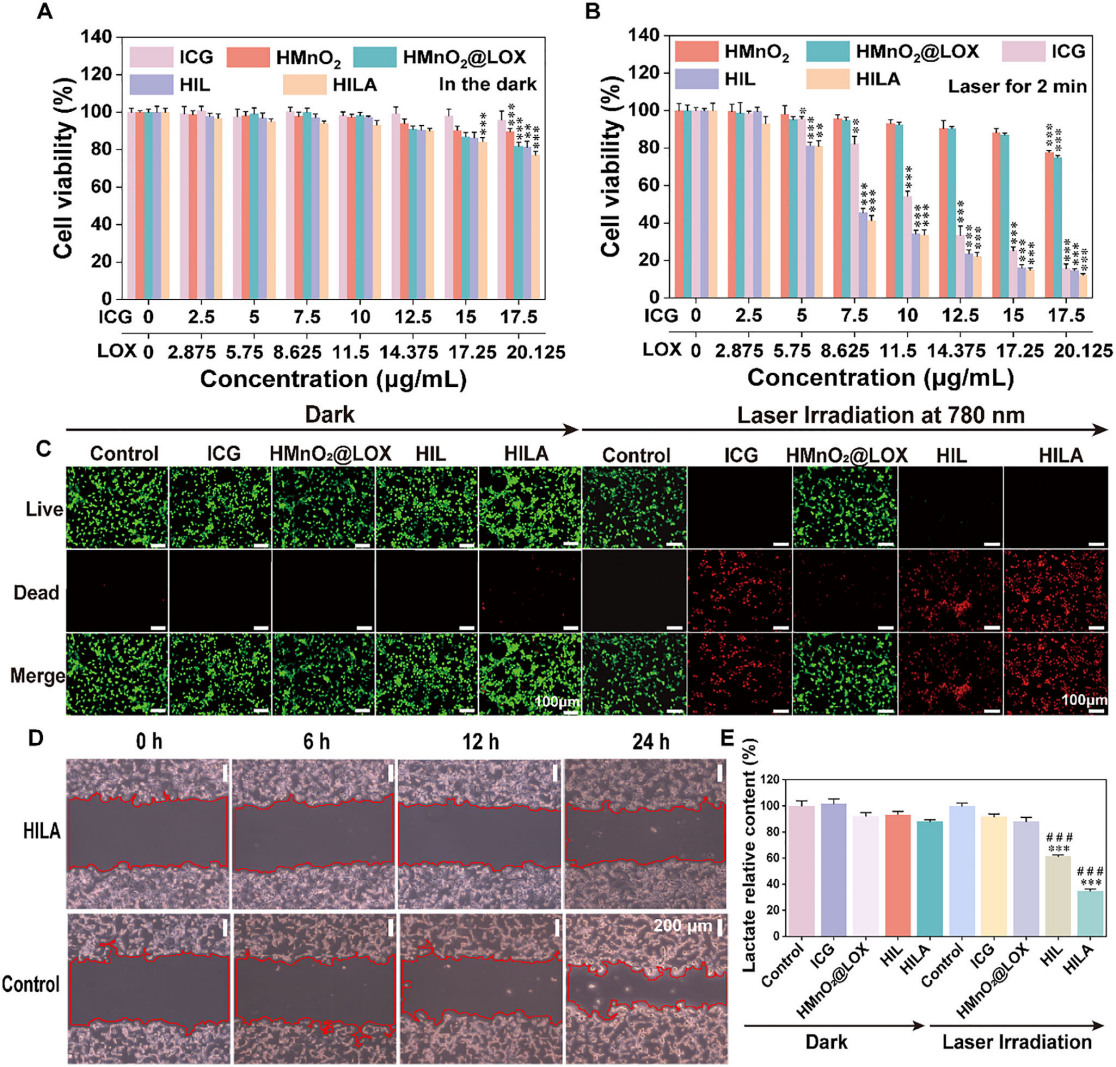
In Vitro Cytotoxicity. (A, B) Cytotoxicity of different formulations against CT26 cells under dark conditions (A) and NIR laser irradiation (B). (C) Live/dead cell staining images indicating cell viability and death status in different groups (scale bar = 100 µm). (D) Scratch assay images demonstrating the migration inhibition ability of HILA at 0, 6, 12, and 24 h (scale bar = 200 µm). (E) Quantitative analysis of intracellular lactate levels in CT26 cells after various treatments. Data are presented as mean ± SD (*n* = 5). Statistic significances between every two groups was determined by *two‐tailed Student's t‐test*. ^*^ *p* < 0.05, ^**^ *p* < 0.01, and ^***^ *p* < 0.001 vs. the control group or 0 µg/mL group. ^#^ *p* < 0.05, ^##^ *p* < 0.01, and ^###^ *p* < 0.001 vs. the irradiated control group.

Furthermore, the contributions of starvation therapy, CDT, and PDT/PTT of HILA to the overall therapeutic efficacy were quantified based on cell viability data obtained at an ICG concentration of 20 µg/mL in the dark and 10 µg/mL under laser irradiation (Figure S22). In the dark, free ICG showed no lethality, and HILA's efficacy was primarily driven by CDT‐ and LOX‐mediated starvation therapy (Figure S22A), with contributions of 59.7% and 40.3%, respectively (Figure S22C); HA‐mediated tumor targeting further enhanced this synergistic effect. Upon laser irradiation, the therapeutic mode shifted to be dominated by ICG‐mediated PDT/PTT, with CDT and starvation therapy acting as adjuvants (Figure S22B). Specifically, PDT/PTT contributed approximately 72.6%, while CDT and starvation therapy contributed 17.3% and 10.1%, respectively (Figure S22D). These quantitative results confirm that under laser activation, phototherapy becomes the predominant mode, while CDT and LOX synergistically enhance the overall efficacy by remodeling the tumor microenvironment.

To further clarify the cytotoxicity of HILA, we used a live/dead cell staining kit to qualitatively assess the viability of CT26 cells under different treatment conditions. As shown in Figure 5C, the blank control showed negligible cell death under both dark and irradiated conditions (1 W/cm^2^, 2 min), indicating that the laser conditions themselves had no significant effect on cell status. The HILA group (containing 17.5 µg/mL of ICG and 20.125 µg/mL of LOX) exhibited faint red fluorescence in the dark, indicative of basal cell death via starvation/CDT. Crucially, irradiated HILA displayed the strongest red fluorescence, signifying superior cell killing. This result underscores the multi‐modal synergy: ICG‐mediated PDT/PTT is amplified by the O_2_/H_2_O_2_ generated from the HMnO_2_‐LOX cycle, while Mn^2+^‐driven CDT and starvation therapy further synergize to maximize antitumor efficacy.

#### 2.5.3 In Vitro Lactate Consumption and Tumor Cell Migration

Lactate serves as a critical energy source and signaling molecule in the TME, driving metabolic reprogramming, tumor growth, and metastasis [43]. The in vitro lactate consumption capacity of HILA was assessed using a lactate detection kit. As shown in Figure 5E, the blank control exhibited no significant difference in lactate levels between dark and irradiated conditions, confirming that laser exposure alone does not affect intracellular lactate content. Free ICG showed negligible impact in the dark but a slight reduction upon irradiation, suggesting that PDT/PTT‐induced cytotoxicity partially affects lactate metabolism. In contrast, formulations containing HMnO_2_@LOX demonstrated pronounced lactate depletion, particularly under laser irradiation. Notably, the HILA group exhibited the most robust effect, reducing intracellular lactate by approximately 60% under irradiation. Mechanistically, this superior efficacy stems from the self‐sustaining HMnO_2_‐LOX catalytic cycle. LOX catalyzes the conversion of lactate into H_2_O_2_ and pyruvate, while HMnO_2_ decomposes H_2_O_2_ to replenish O_2_, establishing a continuous loop for lactate consumption and hypoxia alleviation [44, 45]. Furthermore, the localized hyperthermia generated by ICG under irradiation accelerates this catalytic activity, thereby amplifying lactate depletion. Consequently, the anti‐metastatic potential of HILA was evaluated via scratch assays (Figure 5D and Figure S23). In the dark, the HILA group significantly suppressed cell migration (19.92% vs. 50.30% in control) at 24 h. Under NIR irradiation (1 W/cm^2^, 2 min), HILA induced rapid cell shrinkage and detachment (Figure S24), indicating potent cytotoxicity that precluded migration assessment. These findings confirm that HILA effectively depletes lactate to induce starvation and inhibit metastasis, with efficacy further augmented by ICG‐mediated PDT/PTT.

### 2.6 Investigation of Cell Death Mechanisms

The potent antitumor effect of HILA is driven by its self‐sustaining catalytic cycle within the TME, which triggers a ROS storm by the synergy of PDT/PTT and CDT. In addition, the degradation of HILA consumes GSH, a critical antioxidant, thereby exacerbating ROS accumulation. To elucidate the underlying cell death mechanisms, intracellular ROS levels and GSH content were systematically evaluated.

#### 2.6.1 Cellular ROS Detection

Intracellular ROS generation in CT26 cells was quantitatively assessed using DCFH‐DA fluorescence probing (Figure 6A). In the dark, only the HILA group exhibited significant ROS generation over a 4‐h duration, attributed to the self‐sustaining catalytic cycle and Mn^2+^‐mediated CDT, which was further enhanced by HA‐facilitated endocytosis. Under NIR irradiation, while control formulations (ICG, HIL) exhibited faint green fluorescence, the HILA group exhibited the most intense green fluorescence. This confirms that HA modification significantly promotes nanoparticle uptake, thereby maximizing ROS generation through combined PDT/PTT and CDT effects.

**FIGURE 6:**
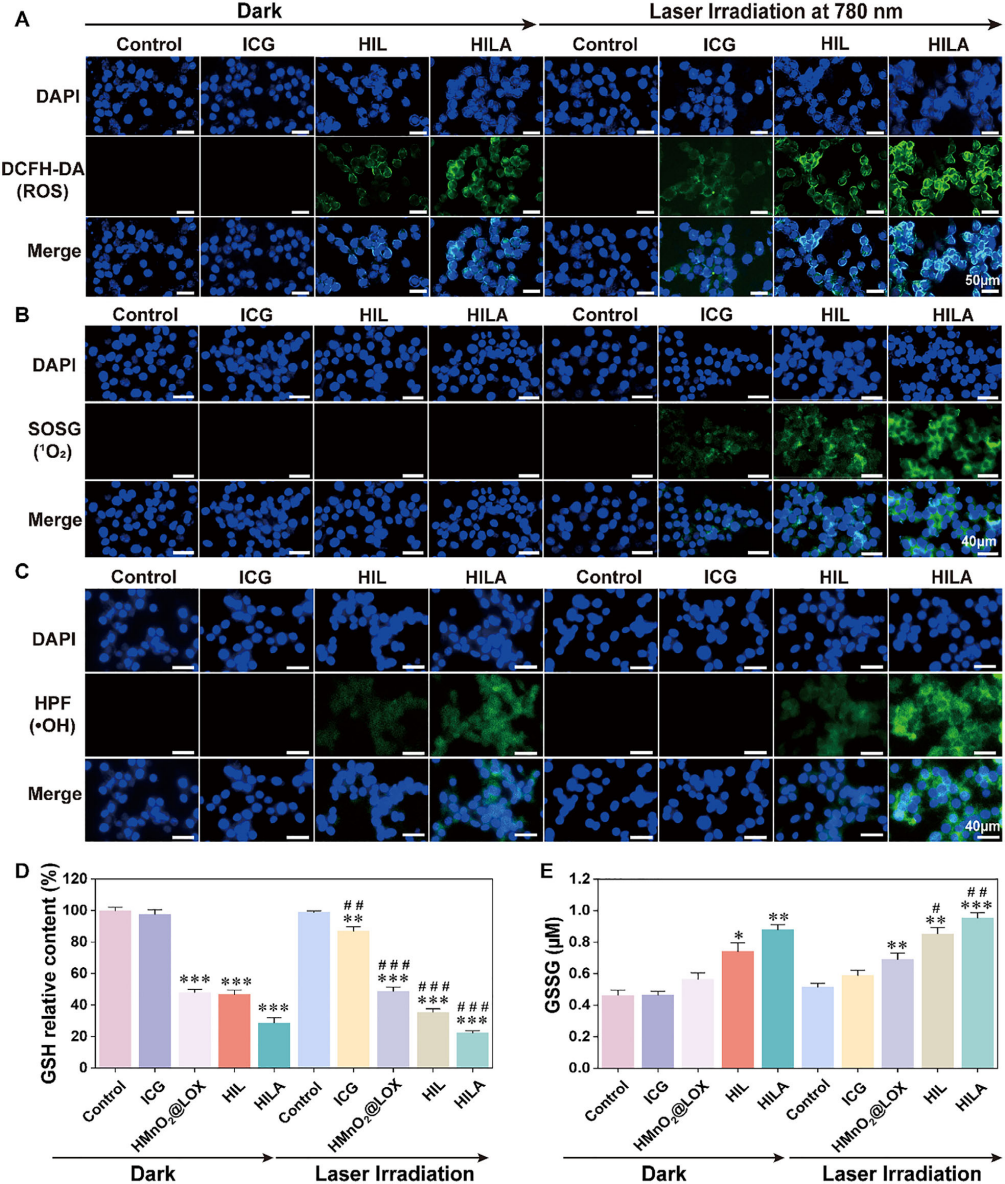
Investigation of Cell Death Mechanisms. (A) Representative fluorescence images of intracellular ROS detected by DCFH‐DA probe (scale bar = 50 µm). (B) Fluorescence image of ^1^O_2_ generation detected by SOSG probe (scale bar = 40 µm). (C) Fluorescence image of •OH generation detected by HPF probe (scale bar = 40 µm). (D) Quantitative analysis of intracellular GSH levels. (E) Quantitative analysis of intracellular GSSG levels. Data are presented as mean ± SD (*n* = 3). Statistic significances between every two groups were determined by *two‐tailed Student's t‐test*. ^*^ *p* < 0.05, ^**^ *p* < 0.01, and ^***^ *p* < 0.001 vs. the control group. ^#^ *p* < 0.05, ^##^ *p* < 0.01, and ^###^ *p* < 0.001 vs. the irradiated control group.

To further elucidate the specific ROS sources, the ^1^O_2_ generated by PDT of ICG and the •OH generated by CDT were qualitatively detected. ^1^O_2_ generation was monitored using SOSG fluorescence assay (Figure 6B). No detectable ^1^O_2_ signals were observed across treatment groups under dark conditions. Following photoactivation, ICG‐containing formulations exhibited significant ^1^O_2_‐specific fluorescence, with HILA showing the strongest signal. This is attributed to in situ O_2_ regeneration by the HMnO_2_‐LOX cycle, which alleviates hypoxia and maintains substrate availability for ICG‐mediated PDT. Furthermore, HA surface modification promotes cellular uptake of the formulation, leading to increased intracellular concentration and improved PDT efficacy of ICG.

•OH generation was tracked using the hydroxyphenyl fluorescein (HPF) probe (green fluorescence in Figure 6C). Under dark conditions, detectable •OH signals were confined to HMnO_2_‐containing formulations (HIL and HILA), with HILA exhibiting greater intensity than HIL. This confirms Mn^2+^‐mediated Fenton‐like catalysis through HMnO_2_ decomposition in the remodeling TME. Additionally, HA functionalization amplified •OH production by enhancing nanoparticle internalization. Notably, irradiation significantly augmented •OH yields in both HIL and HILA groups. This suggests that ICG‐derived photothermal acceleration of the reaction kinetics of Mn^2+^‐mediated Fenton‐like catalysis, which is consistent with the results of in vitro assays. This photothermal‐chemodynamic synergy establishes HILA as a stimuli‐responsive •OH amplification platform overcoming intrinsic limitations of CDT in TME.

#### 2.6.2 Determination of Intracellular GSH Content

Intracellular GSH, a pivotal antioxidant safeguarding cellular components against oxidative damage [46], was quantified to evaluate redox homeostasis disruption (Figure 6D,E). HMnO_2_‐containing formulations (HMnO_2_@LOX, HIL, and HILA) significantly depleted GSH reserves independent of irradiation, confirming structure‐dependent consumption. Notably, HILA induced maximal GSH depletion (approximately 75% decrease vs. untreated control), concurrently elevating oxidized glutathione (GSSG) levels, indicative of irreversible antioxidant exhaustion. Moreover, irradiated free ICG induced moderate GSH reduction, likely due to ^1^O_2_ generation and PTT‐induced metabolic disruption [47, 48]. These results demonstrate that HILA effectively collapses the intracellular antioxidant defense, amplifying oxidative stress to exert potent cytotoxicity.

Collectively, HILA nanoparticles orchestrate a self‐amplifying ROS cascade within the TME through CDT/PDT/PTT synergistic mechanisms, combined with GSH consumption via nanoparticle degradation and targeted delivery optimization by HA surface functionalization, overcoming intrinsic TME barriers (hypoxia, GSH overexpression, poor biodistribution) to elicit a lethal ROS storm, establishing HILA as a theranostic platform for precision tumor ablation.

### 2.7 Biocompatibility and In Vivo Antitumor Efficacy

To ensure the safety and efficacy of HILA in biomedical applications, we systematically evaluated its biocompatibility and in vivo antitumor performance, which form a crucial foundation for translating its outstanding in vitro antitumor effects into clinical practice. The results of hemolysis assays (Figure S25) demonstrated exceptional blood compatibility (<5% hemolysis across all formulations), confirming safety for intravenous administration. Additionally, pharmacokinetic studies (Figure 7B and Table S4) revealed that the HILA maintained higher blood concentrations over 24 h, with significantly prolonged mean residence time (MRT_0‐t_), half‐life (t_1/2_), and area under the concentration‐time curve (AUC_0‐t_). This indicates its long‐circulation characteristics, significantly enhancing ICG bioavailability, prolonging in vivo retention time, and enabling anti‐tumor effect.

**FIGURE 7:**
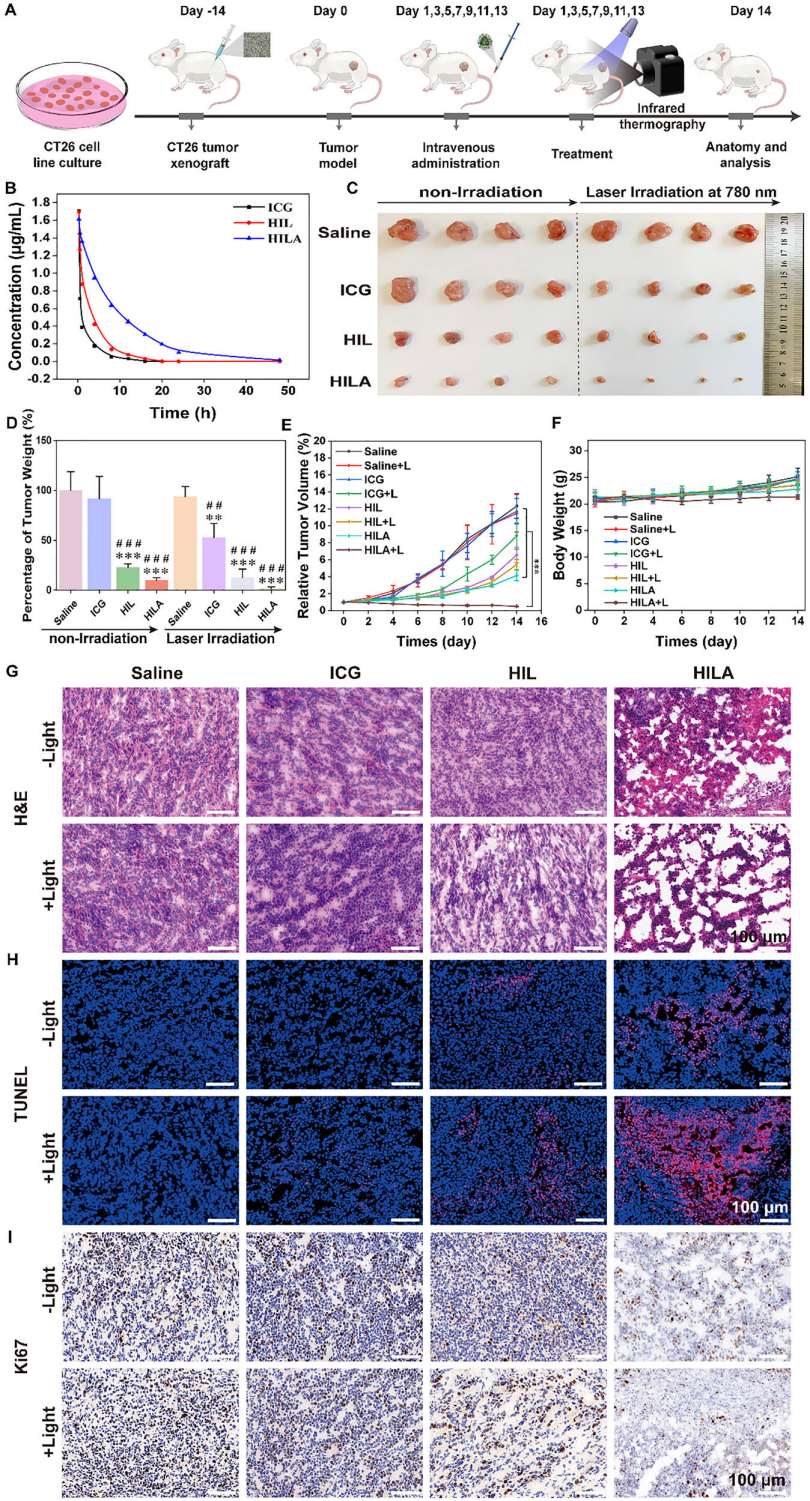
Biocompatibility and In Vivo Antitumor Efficacy. (A) Schematic illustration of the establishment of the CT26 tumor‐bearing mouse model and the in vivo treatment protocol. (B) Plasma concentration‐time curves of free ICG, HIL, and HILA. (C) Representative photographs of tumors excised from different groups at the end of the study. (D) Average tumor weights and (E) tumor growth curves recorded during the treatment period. (F) Body weight change curves for the mouse in each treatment group. (G) H&E, (H) TUNEL, and (I) Ki67 immunohistochemical staining of tumor tissue sections from each treatment group (scale bar = 100 µm). Data are presented as mean ± SD (*n* = 5). Statistic significances between every two groups was determined by *two‐tailed Student's t‐test*. ^*^ *p* < 0.05, ^**^ *p* < 0.01, and ^***^ *p* < 0.001 vs. the saline group. ^#^ *p* < 0.05, ^##^ *p* < 0.01, and ^###^ *p* < 0.001 vs. the saline+L group.

The in vivo therapeutic effect of HILA was evaluated in CT26 tumor‐bearing mice (Figure 7A), and the dosing frequency was determined based on preliminary pharmacokinetic data (Table S4). Mice were divided into eight groups (5 per group) and administered intravenously with saline, ICG (10 mg/kg), HIL, or HILA (calculating with ICG, 10 mg/kg), with or without NIR irradiation (780 nm, 2 W/cm^2^). As shown in Figure 7C–E, ICG‐containing formulations significantly inhibited tumor growth under irradiation. Notably, the HILA group exhibited the most potent antitumor activity, achieving near‐complete tumor suppression upon laser exposure, whereas laser irradiation alone had negligible impact. Ex vivo tumor weights (Figure 7D) corroborated these findings, showing the lowest tumor weight in the HILA group, with further reductions observed after laser treatment, consistent with the tumor volume data (Figure 7E). Throughout the treatment period, no significant body weight fluctuations were observed (Figure 7F), indicating minimal systemic toxicity. Histological analysis provided mechanistic insights. Hematoxylin and eosin (H&E) and TUNEL staining (Figure 7G,H) revealed extensive apoptosis and tissue destruction in the HILA group. Notably, H&E images of the HILA+L group (Figure S26) showed extensive air‐filled cavities, serving as morphological evidence of tissue collapse post‐treatment [49]. Immunohistochemical analysis showed significantly reduced Ki67 expression in HILA‐treated tumors (Figure 7I and Figure S27A), confirming effective proliferation inhibition via lactate depletion. These therapeutic outcomes are primarily attributed to the self‐sustaining catalytic cycle of HILA, resulting in lactate depletion and remodeling the TME, thereby enhancing Mn^2+^‐mediated CDT as well as ICG‐driven PDT/PTT effects. This synergistic mechanism promotes efficient ROS generation and GSH consumption, enabling continuous tumor cell killing.

Furthermore, the in vivo biosafety of nanoparticles was evaluated at the experimental endpoint. Comprehensive blood biochemistry analyses (Figure S27) revealed no significant differences between the nanoparticle‐treated group and the control groups 14 days postadministration, indicating that HILA did not induce systemic toxicity under experimental conditions. Concurrently, histological examination of major organs, including the heart, liver, spleen, lungs, and kidneys across all groups (Figure S28) revealed no evident tissue damage or pathological abnormalities compared to the control group, demonstrating the excellent biocompatibility and biosafety of the nanoparticles. Collectively, HILA demonstrates a superior safety profile while orchestrating synergistic starvation, CDT, PDT, and PTT for efficient tumor eradication.

## 3 Conclusions

In summary, we successfully developed a self‐amplifying nanoplatform (HILA) that orchestrates tumor‐specific cascading reactions to overcome microenvironmental barriers in colorectal cancer. In HILA, LOX‐mediated lactate oxidation depletes metabolic substrates (inducing starvation therapy) while simultaneously acidifying the TME and generating H_2_O_2_. This H_2_O_2_ fuels HMnO_2_ decomposition into O_2_ (alleviating hypoxia) and cytotoxic •OH (enhancing CDT), establishing a self‐sustaining HMnO_2_‐LOX catalytic cycle that continuously consumes lactate and remodels TME. Concurrently, under NIR irradiation, ICG released by HILA degradation in acidic and GSH‐rich TME generates ^1^O_2_ for PDT and hyperthermia for PTT, which in turn enhancs the cascade catalytic cycle. Crucially, this self‐propelling catalytic cycle creates a positive feedback loop that perpetually depletes lactate, remodels TME, and amplifies starvation therapy, CDT, PDT, and PTT synergistic efficacy for eradication of colon cancer. The HA modification guarantees the biocompatibility and targeted tumor delivery of HILA, further enhancing the quad‐model synergistic therapy. This work pioneers a paradigm of cascade‐driven precision therapy, offering a clinically translatable blueprint for treating minimally invasive cancer.

## 4 Experimental Section

### 4.1 Materials and Instruments

#### 4.1.1 Materials

HMnO_2_ (100 nm in TEM) was purchased from Xi'an Qiyue Biotechnology Co., Ltd. ICG, DL‐lactate, 3‐aminopropyltrimethoxysilane (APTMS), dimethyl sulfoxide (DMSO), and 1,3‐diphenylisobenzofuran (DPBF) were purchased from Shanghai Macklin Biochemical Technology Co., Ltd. Singlet oxygen fluorescence green (SOSG) was purchased from Dalian Meilun Biotechnology Co., Ltd. Hydroxyphenyl fluorescein (HPF) was purchased from Shanghai Maokang Biotechnology Co., Ltd. LOX, HA (Mw 3000), GSH, and MB were purchased from Shanghai Aladdin Biochemical Technology Co., Ltd. DMEM medium, streptomycin/penicillin antibiotics, fetal bovine serum (FBS), and trypsin were provided by Wisent Inc. DAPI and Hoechst 33342 were purchased from Shanghai Myrell Chemical Technology Co., Ltd. The BCA protein concentration assay kit, high‐efficiency RIPA tissue/cell lysis buffer, and PMSF were purchased from Beijing Solarbio Science & Technology Co., Ltd. Coumarin 6 (C6), 2',7'‐Dichlorofluorescein diacetate (DCFH‐DA), and Lactate Detection Kit were provided by Wuhan Abbkine Biotechnology Co., Ltd. GSH and GSSG Detection Kits were provided by Shanghai Biyun Tian Biotechnology Co., Ltd. Live/Dead Cell Staining Kit was provided by Yakein Biotechnology Co., Ltd. Mouse colon cancer CT26 cells were obtained from Professor Linghu Kegang's laboratory.

SPF‐grade 9 female SD rats (6‐week‐old, weighing 200–220 g) and 80 female BALB/C mice (4‐week‐old, weighing 20–22 g) purchased from Beijing Fukang Biotechnology Co., Ltd., License No.: SCXK(Jing)2024‐0003. Both rats and mice were housed in individual cages under conditions of 20–26°C and 50%–60% relative humidity, with free access to food and water. Animals exhibited good health status. Prior to experiments, mice underwent a one‐week acclimation period under standard conditions following Institutional Animal Care and Use Committee (IACUC) guidelines. All animal experiments were reviewed and approved by the IACUC at Guizhou Medical University (Approval number: 2400344).

#### 4.1.2 Instruments

Thermal imaging of formulations and animals was captured using an infrared thermal imager (Beijing Hongpu Weishi Image Technology Co., Ltd., China). In vitro laser irradiation interventions were performed using a 780 nm laser (Jilin Mingwan Scientific Instrument Co., Ltd., China). Particle size, zeta potential, and polydispersity index (PDI) were measured using a Nano‐Zetasizer and Zeta Potential Analyzer (Bruker‐Hewlett, New York, USA). Particle morphology was observed via transmission electron microscopy (TEM, FEI Talos 200S, Thermo Fisher Scientific, USA). Elemental information of the formulations was analyzed using an X‐ray Photoelectron Spectroscopy (XPS, Thermo K‐Alpha, America) and a Fourier Transform Infrared Spectrometer (FTIR‐650, Tianjin Gangdong Technology Development Co., Ltd., China). Absorbance or spectral measurements were performed using a UV–Vis Spectrophotometer (UV‐2700, Shimadzu Corporation, Japan) and a Microplate Reader (Thermo Fisher Scientific, Waltham, USA). Fluorescence in cells was quantified using a NoVoCyt flow cytometer (EisenBio Hangzhou Co., Ltd., China). Fluorescence in cells or tissues was observed using an inverted fluorescence microscope (XDS‐2B, Nikon Corporation, Japan). The biodistribution of formulations in mice was analyzed using an IVIS in vivo imaging system (PerkinElmer, USA).

### 4.2 Methods

#### 4.2.1 Preparation of HILA

HMnO_2_ nanoparticles (2.5 mg) were first dispersed in 1 mL of ethanol and aminated with APTMS (0.5 µL) via ultrasonication and shaking for 1 h each. Subsequently, LOX was covalently immobilized onto the aminated HMnO_2_ through an amide reaction by mixing both components (3 mg/mL) and shaking in the dark for 24 h. The product, HMnO_2_@LOX, was collected by centrifugation (12 000 rpm, 5 min). ICG was then loaded by incubating HMnO_2_@LOX with an ICG solution (6 mg/mL) for 12 h in the dark, followed by centrifugation and washing to yield HIL. Finally, HIL was coated with HA by shaking with an HA solution (2 mg/mL) for 12 h in the dark, and the resulting HILA nanoparticles were obtained via centrifugation (12 000 rpm, 10 min).

#### 4.2.2 Evaluation of In Vitro Catalytic Performance of HILA

##### 4.2.2.1 Detection of H_2_O_2_/O_2_ Generation from The HMnO_2_–LOX Catalytic Cycle

To evaluate LOX activity, HILA and control formulations (HIL, LOX, and HMnO_2_, LOX‐equivalent concentration: 100 µg/mL) were incubated with lactate (5 mg/mL) for 30 min, followed by H_2_O_2_ quantification using a commercial kit. Catalytic kinetics were further assessed by measuring H_2_O_2_ generation from HILA in response to varying lactate concentrations (2–20 mg/mL). For HMnO_2_ catalytic activity assessment, oxygen evolution was first qualitatively assessed by photographing bubble formation upon reacting HMnO_2_ (1 mg/mL) or HILA (500 µg/mL) with H_2_O_2_ (25 mM). Subsequently, dissolved oxygen kinetics were quantified in real‐time using an oxygen electrode for HILA (0–50 µg/mL) incubated with H_2_O_2_. Finally, the efficiency of the self‐sustaining catalytic cycle was validated by comparing O_2_ consumption between HILA and free LOX (200 µg/mL LOX‐equivalent) in the presence of lactate (40 mM).

##### 4.2.2.2 Degradation Property and ICG Release Profiling of HILA in TME

The degradation dynamics of HILA nanoparticles were evaluated by monitoring ICG release under simulated TME conditions, quantified via UV–Vis spectrophotometry at 780 nm. For pH‐responsive release studies, HILA (1 mg/mL) was incubated in PBS at varying pH values (5.0, 6.8, 7.4) at 37°C for 40 h. At predetermined time intervals, the supernatant was collected for analysis and replenished with an equal volume of fresh PBS. Parallel experiments assessed ICG release in the presence of GSH (0–10 mM) or H_2_O_2_ (0–5 mM) over a 6 h period. To investigate the impact of photothermal effects, release profiles were first evaluated at 42°C under different pH conditions. Furthermore, NIR‐triggered release was assessed by subjecting HILA samples to NIR irradiation (780 nm, 1 W/cm^2^, 5 min) at 45 min intervals during incubation.

##### 4.2.2.3 Detection of •OH Generation in Mn^2+^‐Catalyzed H_2_O_2_ Decomposition

•OH generation was assessed by monitoring the oxidative degradation of MB, indicated by the decay of its characteristic absorption at 665 nm. To evaluate GSH‐responsive •OH production, HILA and control formulations (ICG, HMnO_2_, HIL; LOX‐equivalent concentration: 100 µg/mL) were incubated in NaHCO_3_ buffer with graded GSH concentrations (0–10 mM) at 37°C. Following the addition of H_2_O_2_ (10 mM) for 30 min, MB (10 µg/mL) was introduced for 1.5 h, after which absorbance was measured. To investigate lactate‐driven cascade catalysis, HILA (100 µg/mL) was incubated with lactate (2.5 mg/mL), H_2_O_2_ (10 mM), and MB (5 µg/mL) in buffer under continuous shaking at 37°C overnight, followed by absorbance analysis. Additionally, the pH‐dependent (pH 5.0 vs. 7.4) and NIR‐responsive (780 nm, 1 W/cm^2^, 5 min) catalytic performances of HILA were systematically evaluated using the same MB oxidation protocol.

#### 4.2.3 Evaluation of HILA's Photodynamic Effects and Photothermal Capabilities

##### 4.2.3.1 Detection of ^1^O_2_ Generation

The ^1^O_2_ generation capability of HILA nanoparticles under NIR irradiation was systematically evaluated using DPBF decay and SOSG fluorescence assays. For DPBF measurements, HILA and control formulations (ICG‐equivalent concentration: 10 µg/mL) were mixed with DPBF (0.1 µM). Under NIR irradiation (780 nm, 1 W/cm^2^, 5 min), ^1^O_2_ production was monitored in real‐time by recording the absorbance decay of DPBF at 412 nm at 1‐min intervals. To simulate the TME and assess lactate‐driven enhancement, 40 mM lactate was introduced into the HILA suspension, and ^1^O_2_ generation was quantified using the same DPBF protocol. Additionally, to validate H_2_O_2_‐driven oxygen supply enhancement, HILA (equivalent concentration: ICG 10 µg/mL) was incubated with 500 µM H_2_O_2_ and compared to controls using the SOSG probe (10 µM). Following NIR irradiation, ^1^O_2_ generation was quantified by measuring SOSG fluorescence intensity (Ex: 504 nm, Em: 525 nm) using a microplate reader.

##### 4.2.3.2 Evaluation of Photothermal Effect

The photothermal properties of HILA were systematically evaluated against control formulations (HMnO_2_, HMnO_2_@LOX, HMnO_2_@ICG, HIL, and free ICG; ICG‐equivalent concentration: 10 µg/mL). Samples (1 mL) were subjected to NIR laser irradiation (780 nm, 1 W/cm^2^, 5 min), and temperature variations were monitored in real‐time using an infrared thermal camera. Concentration‐dependent photothermal effects were further investigated using HILA solutions ranging from 25 to 150 µg/mL. Photothermal stability was assessed over three consecutive laser on/off cycles. Additionally, to investigate the self‐sustaining catalytic cycle within the TME, the photothermal performance of HILA was compared between lactate‐rich (40 mM) and normal conditions, highlighting the impact of lactate‐driven oxygen generation on ICG‐mediated PTT.

#### 4.2.4 Cellular Uptake and Targeting of HILA

CT26 cells were seeded in 12‐well plates (2 × 10^5^ cells/well) and cultured for 48 h to reach optimal confluence. Subsequently, cells were treated with fresh medium containing ICG, HIL, or HILA (each at a concentration of 20 µg/mL) for 1, 2, or 4 h. After incubation, cells were washed three times with PBS, stained with Hoechst 33342 (1 mL/well, 30 min, dark) to label nuclei, and imaged using fluorescence microscopy to evaluate nanoparticle internalization. To validate HA‐mediated CD44 targeting, cells were pre‐treated with free HA (0.25 mM) for 1 h to block surface receptors, followed by washing and incubation with HILA (20 µg/mL, 4 h). Nuclear staining and fluorescence imaging were then performed to compare cellular uptake with that of un‐pretreated controls. For quantitative analysis, coumarin‐6 (C6)‐labeled HIL@C6 and HILA@C6 were prepared, and cellular uptake was quantified by flow cytometry.

#### 4.2.5 Live‐Dead Cell Staining Experiment

CT26 cells were seeded in 12‐well plates (3 × 10^5^ cells/well) and cultured for 48 h. Subsequently, cells were then incubated with fresh medium containing ICG, HIL, or HILA (20 µg/mL) for 4 h (*n* = 2 replicates per group). After incubation, the cells were washed three times with PBS and replaced with fresh culture medium. One replicate per group received NIR irradiation (780 nm, 1 W/cm^2^, 2 min), while the corresponding control replicate was kept in darkness. Following an additional 12‐h incubation period, cells were stained with Calcein‐AM (8 µM, live cells; λex/λem: 488/510 nm) and propidium iodide (PI, 2 µM, dead cells; λex/λem: 535/617 nm) for 30 min. After staining, cells were washed three times with PBS and subjected to fluorescence microscopy imaging to assess cell viability.

#### 4.2.6 Wound Healing Assay for Cell Migration Evaluation

CT26 cells were seeded in 6‐well plates and cultured to 90%–100% confluence. A uniform scratch was created using a sterile 200 µL pipette tip, followed by two washes with PBS to remove detached cells and debris. Cells were divided into two groups and incubated with either standard medium containing 1% FBS or medium supplemented with HILA (20 µg/mL) under standard culture conditions (37°C, 5% CO_2_). Wound closure was monitored at predetermined time points (0, 6, 12, and 24 h) using phase‐contrast microscopy, with images captured at identical positions for quantitative analysis.

#### 4.2.7 Intracellular Lactate and GSH Quantification Assay

##### 4.2.7.1 Lactate Assay

CT26 cells were seeded in 12‐well plates and cultured for 48 h prior to treatment with ICG, HMnO_2_@LOX, HIL, or HILA nanoparticles (20 µg/mL). Cells were assigned to either laser‐treated groups (following 4 h of incubation and irradiation at 780 nm NIR, 1 W/cm^2^ for 2 min) or non‐irradiated control groups. After an additional 10 h incubation, cells were washed with ice‐cold PBS, pelleted by centrifugation (1000 rpm, 5 min, 4°C), and lysed in Lactate Assay Buffer (1 mL per 5 × 10^6^ cells) via ice‐bath sonication (20% amplitude, 200 W, 3 s on/7 s off, 30 cycles). Lysates were centrifuged (12 000 g, 5 min, 4°C), and supernatants were analyzed using the CheKine Lactate Assay Kit (KTB1100, Abbkine, Lot No. ATYA24101).

##### 4.2.7.2 GSH/GSSG Assay

Cell pellets from parallel treatments were resuspended in three times the volume of Protein Removal Reagent M, vortexed thoroughly, and subjected to two rapid freeze‐thaw cycles (alternating between liquid nitrogen and a 37°C water bath). After 5 min incubation on ice, lysates were centrifuged (10 000 g, 10 min, 4°C). Supernatants were assayed for total GSH and GSSG using a GSH/GSSG Detection Kit (S0053, Beyotime, Lot No. 061923230841), with absorbance measured at 412 nm (GSH) and 340 nm (GSSG) via a microplate reader.

#### 4.2.8 Intracellular ROS Detection

To systematically evaluate nanoparticle‐induced oxidative stress, CT26 cells were seeded in 12‐well plates (2 × 10^5^ cells/well) and cultured for 48 h prior to treatment with ICG, HMnO_2_@LOX, HIL, or HILA nanoparticles (20 µg/mL) for 4 h. Subsequently, cells were divided into laser‐treated (780 nm, 1 W/cm^2^, 2 min) and non‐irradiated groups. Intracellular ROS generation was assessed using three complementary probes. Total ROS levels were determined by loading cells with DCFH‐DA (10 µM, 30 min), followed by counterstaining with DAPI (10 µg/mL) and imaging via fluorescence microscopy (λex/λem: 488/525 nm). ^1^O_2_ production was evaluated in parallel samples using SOSG (5 µM, 30 min), and ^1^O_2_‐specific fluorescence was measured at λex/λem: 504/525 nm. •OH levels were detected using HPF (5 µM, 30 min), with fluorescence signals recorded at λex/λem: 490/515 nm.

#### 4.2.9 In Vivo Biodistribution Study

The 36 female BALB/c mice bearing CT26 tumors (10^6^ cells/mouse) were randomized into 3 groups (*n* = 12). At predetermined timepoints (2, 4, 8, 24 h), mice per group received intravenous injections of free ICG, HIL, or HILA (ICG‐equivalent 10 mg/kg in 100 µL saline) via the tail vein after alcohol disinfection. Real‐time nanoparticle distribution was monitored using the IVIS in vivo imaging system (785–840 nm). Post‐ussacrifice, major organs (heart, liver, spleen, lungs, kidneys) and tumors were excised for ex vivo fluorescence imaging to quantify ICG accumulation.

#### 4.2.10 In Vivo Antitumor Efficacy Evaluation

The tumor‐bearing BALB/c mice were randomly divided into 8 groups (*n* = 5) and received intravenous injections via the tail vein on days 0, 1, 3, 5, 7, 9, 11, and 13 with saline (control), ICG (10 mg/kg), HIL, or HILA (dosed at 10 mg/kg ICG‐equivalent). For in vivo studies, a higher power density of 2 W/cm^2^ was selected to compensate for tissue‐induced laser attenuation. Within each treatment group, half of the animals were subjected to near‐infrared irradiation (780 nm, 2 W/cm^2^, 5 min) at 4 h and 24 h postinjection, while the other half served as non‐irradiated controls. Tumor volume (caliper measurement) and body weight were recorded every 2 days. On day 14 (1 day after the final administration), mice were euthanized, and blood samples, tumor tissues, and major organs were collected. Tumors from each treatment group were weighed and photographed. Major organs (including the heart, liver, spleen, lung, and kidney) were fixed in 4% paraformaldehyde, processed for dehydration, embedded in paraffin, sectioned, and stained with hematoxylin and eosin (H&E) for histopathological evaluation. Tumor tissues underwent similar fixation and processing and were subjected to immunohistochemical staining for Ki67, H&E staining, and TUNEL assay for apoptosis detection. All tissue sections were examined and analyzed under an Olympus BX‐51 microscope. Blood samples were used for complete blood count and serum biochemical analysis.

#### 4.2.11 Statistical Analysis

Data are presented as mean ± SD. Normality and homogeneity of variance were tested prior to analysis. Outliers were assessed using *Grubbs’ test*, and none were identified for exclusion. For in vitro experiments, at least three independent replicates were performed (*n* ≥ 3). For in vivo studies, each group contained a minimum of five mice (*n* = 5). Two‐group comparisons used Student's *t*‐test, multi‐group analyses employed *one‐way ANOVA* with significance thresholds: ^*^ *p *< 0.05, ^**^ *p *< 0.01, ^***^ *p *< 0.001, *ns* indicates no statistical significance, and all data met the assumptions of normality and homogeneity of variance. All statistical analyses were conducted using GraphPad Prism 10.0 or Origin 2019b.

#### 4.2.12 Declaration of Generative AI and AI‐Assisted Technologies in the Writing Process

We employed DeepSeek and Doubao AI to assist with text polishing and the conceptual optimization of figure illustrations, while Grammarly and Ginger were used for English grammar checking and language refinement. The use of these tools was strictly limited to language optimization and aiding the conceptualization of the figures. All scientific concepts, the interpretation of experimental data, the derivation of conclusions, and the core academic viewpoints were independently developed by the authors, who assume full responsibility for the entire content of the manuscript.

## Author Contributions

**Shibo Zhao**: methodology, investigation, writing – original draft, writing – review and editing. **Kewu Zeng**: supervision. **Liang Hong**: software. **Xiangyu Zeng**: visualization. **Yuan Li**: validation. **Wenying Wu**: investigation, data curation, writing – review and editing. **Xiangchun Shen**: funding acquisition, supervision. **Zhiwei Duan**: validation. **Chaoda Xiao**: supervision. **Ling Tao**: resources. **Yan Chen**: resources, funding acquisition.

## Conflicts of Interest

The authors declare no conflicts of interest.

## Supporting information




**Supporting File**: advs76367‐sup‐0001‐SuppMat.docx.

## Data Availability

The data that support the findings of this study are available on request from the corresponding author. The data are not publicly available due to privacy or ethical restrictions.
