# Supplementary material for: Self‐Sustaining Lactate Depletion Nanoplatform Remodels Tumor Microenvironment and Augments Synergistic Photodynamic/Photothermal/Chemodynamic/Starvation Therapy for Eradication of Colon Cancer
Source: Adv Sci (Weinh). 2026 Jun 26:e76367. Online ahead of print. doi: 10.1002/advs.76367 (PMC13337002; doi:10.1002/advs.76367)
Supplement: Supplementary file 1 — Supporting File: advs76367‐sup‐0001‐SuppMat.docx. [file ADVS-9999-e76367-s001.docx]

Supporting Information

**Self-sustaining lactate depletion nanoplatform remodels tumor microenvironment and augments synergistic photodynamic/photothermal/chemodynamic/starvation therapy for eradication of colon cancer**

*Yu-E Wang, Shibo Zhao, Wenying Wu, Zhiwei Duan, Yuan Li, Xiangyu Zeng, Liang Hong, Yan Chen, Ling Tao, Kewu Zeng^*^, Chaoda Xiao^*^, Xiangchun Shen^*^*

**1. Supplementary Methods**

**1.1. Characterization of Nanoparticles**

After appropriate dilution of the nanoparticles with deionized water, particle size and zeta potential were measured using a Bruker HAVEN NanoSizer and zeta potential analyzer. Morphology of the nanoparticles was captured under TEM, with samples mounted on 300-mesh copper grids. The mesoporous structure of HMnO_2_ was validated using N_2_ adsorption-desorption method. HMnO_2_ were degassed under vacuum at 150 °C for over 6 h to remove adsorbed impurities. N_2_ adsorption–desorption isotherms were measured at 77 K. The specific surface area and pore size distribution were calculated using the BET and BJH methods, respectively. To assess stability under different conditions, HILA nanoparticles were incubated in 10% FBS, PBS, and DMEM at 37°C. Size variations were monitored at 0, 1, 2, 3, 4, 5, 6, and 7-day intervals using a nanoparticle size analyzer. Fourier Transform Infrared Spectroscopy (FT-IR) was also performed to further determine the structural composition of the nanoparticles. Lyophilized powders (ICG, HMnO_2_, LOX, HMnO_2_+LOX, HMnO_2_+ICG, HIL, and HILA) were compressed into potassium bromide pellets and analyzed using a Fourier Transform Infrared Spectrometer at a resolution of 2 cm^-1^ with 32 scans. To determine the surface elemental composition and chemical state of the nanoparticles, X-ray photoelectron spectroscopy (XPS) analysis was performed. Dried powder samples (including HIL and HILA) were uniformly adhered to conductive adhesive and secured to the sample stage. The tests were conducted on a Thermo Scientific K-Alpha^+^ X-ray photoelectron spectrometer using a monochromatized Al Kα X-ray source (1486.6 eV) with a power of 72 W and a beam spot size of 400 μm. Charge correction was performed using the C 1s peak position (284.8 eV) of contaminated carbon. The full-spectrum scan covered 100 eV with a step size of 1.0 eV.

To determine the drug loading capacity and encapsulation efficiency of ICG in nanoparticles, a standard curve for ICG was first established. Using methyl alcohol as the solvent, a 30 μg/mL ICG stock solution was sequentially diluted to standard solutions of 1, 2, 3, 4, and 5 μg/mL. After determining the maximum absorption wavelength at 780 nm via full-wavelength scanning (300–800 nm), the absorbance of each standard solution concentration was measured at this wavelength. A standard curve was plotted with ICG concentration on the x-axis and absorbance on the y-axis. Sample Preparation: The HILA samples were extracted with methanol by ultrasonic treatment, and the supernatant was obtained by centrifugation. Transfer 200 μL of the supernatant to a 96-well plate and measure absorbance at 780 nm. Perform three parallel measurements and calculate the average value. Calculate the ICG content in the supernatant based on the ICG standard curve. Determine the drug loading (DL) and encapsulation efficiency (EE) using formulas (1) and (2), respectively.

Drug Loading=WtWs×100%*Drug Loading=WtWs×100%*

(1)

Encapsulation Efficiency=WtWt'×100%*Encapsulation Efficiency=WtWt′×100%*

(2)

Where W_t_ is the amount of ICG tested, W_t’_ is the total amount of ICG added, and W_s_ is the total weight of HILA sample.

The BCA protein concentration assay kit was employed to quantitatively analyze the enzyme loading of LOX in HILA. An enzyme-linked immunosorbent assay reader measured the absorbance values at 570 nm for each well, and a BSA standard curve was plotted. Sample preparation: Dilute the centrifuged HILA appropriately and measure its absorbance following the same procedure described above. Three parallel experiments were conducted, and the average value was taken. Calculate the LOX content in HILA based on the BSA standard curve. Use equations (3) and (4) to determine the enzyme loading amount and encapsulation efficiency.

LOX Loading=LOXtestWs×100%*LOX Loading=LOXtestWs×100%*

(3)

Encapsulation Efficiency=LOXtestLOXInputs×100%*Encapsulation Efficiency=LOXtestLOXInputs×100%*

(4)

Where W_s_ is the total weight of HILA sample.

**1.2. Photothermal Conversion Efficiency of HILA**

Further testing of HILA’s photothermal stability was conducted over three cycles of heating and natural cooling. The photothermal conversion efficiency of HILA was calculated using the following formula.

η=hs(Tmax−Tsur)−QsI(1−10−Aλ)*η=hsTmax−Tsur−QsI(1−10−Aλ)*

(5)

τs=mDCDhs*τs=mDCDhs*

(6)

θ=ΔTΔTmax*θ=∆T∆Tmax*

(7)

Qs=mi ci (Tmax−Tsur)t*Qs=mi ci (Tmax−Tsur)t*

(8)

In the equation, h represents the heat transfer coefficient, s denotes the container surface area, T_max_ is the maximum temperature, T_sur_ is the ambient temperature, ∆T_max_ = T_max_ − T_sur_, I is the laser power, A_λ_ is the absorbance of 100 μg/mL HILA at 780 nm. τs is the time constant of the sample system, determined from the linear time data of the sample's natural cooling period relative to -Lnθ. Since the solvent mass far exceeds the solute mass, h_s_ is calculated using pure water mass m_D_ and heat capacity C_D_ (m_D_ = 0.2 g, C_D_ = 4.2 J/g). Q_s_ represents heat loss due to container light absorption, typically measured independently with pure water, where it is the time for water to reach maximum temperature difference.

**1.3. *In Vitro* Cytotoxicity**

*1.3.1. In Vitro Toxicity of HILA on L-929 or CT26 Cells*

The MTT assay was employed to evaluate the *in vitro* cytotoxicity of ICG, HMnO_2_, HMnO_2_@LOX, HIL, and HILA on L-929 cells or colon cancer CT26 cells. Cells were seeded at a density of 6 × 10^3^ cells per well in a 96-well plate and cultured for 24 h to allow attachment. The original medium was then discarded, and fresh medium containing different concentrations (0−30 μg/mL of ICG-equivalent or 0−34.5 μg/mL of LOX-equivalent) of the test samples was added. Culturing continued for another 24 h. After incubation, aspirate the drug-containing medium and gently wash cells three times with PBS to thoroughly remove residual drugs. Add 100 μL fresh medium containing MTT reagent (final concentration 0.05 mg/mL) to each well and incubate for 4 h. Carefully aspirate the supernatant to avoid disrupting the formed MTT crystals. Add 100 μL DMSO to each well and incubate at 37°C with gentle shaking (100 rpm) in the dark for 10 min to ensure complete dissolution of the crystals. Finally, measure the absorbance at 490 nm using a microplate reader and calculate the cell survival rate according to the formula.

Cell viability=As−AbAc−Ab*Cell viability=As−AbAc−Ab*

(9)

As: experimental wells; Ac: control wells; Ab: blank wells.

*1.3.2. Cytotoxicity Testing of HILA Under 780 nm Laser Irradiation*

The MTT assay was employed to evaluate the cytotoxic effects of different formulations on CT26 cells under 780 nm laser irradiation. CT26 cells were seeded at 6×10^3^ cells per well in a 96-well plate and cultured for 24 h to allow cell attachment. After removing the original medium, fresh medium containing different concentrations (0−17.5 μg/mL of ICG-equivalent or 0−20.125 μg/mL of LOX-equivalent) of ICG, HMnO_2_@LOX, HIL, or HILA were added, and cells were cultured for an additional 4 h. Subsequently, discard the drug-containing medium, gently wash the cells three times with PBS (pH 7.4), add 100 μL of fresh medium to each well, and irradiate the light-treated group with 780 nm laser (1 W/cm^2^) for 2 min. Then, continue culturing all groups for 20 h. After incubation, discard the medium, wash three times with PBS, and add 100 μL of fresh medium containing MTT (0.05 mg/mL) to each well. Incubate for 4 h. Carefully aspirate the supernatant. Add 100 μL DMSO to each well. Incubate at 37°C with shaking at 100 rpm for 10 min in the dark to ensure complete dissolution of the MTT crystals. Finally, measure the absorbance at 490 nm and calculate the cell survival rate.

**1.4. Biocompatibility and *In Vivo* Anticancer Efficacy**

*1.4.1. Hemolysis Assay*

To evaluate the biocompatibility of nanoparticles, a hemolysis assay was conducted. Red blood cells (RBCs) were isolated from whole blood of female BALB/c mice. All samples were then centrifuged (3000 rpm, 5 min, 4°C), and the supernatant was measured at 570 nm. Saline and deionized water served as negative and positive controls, respectively.

*1.4.2. Tumor Mouse Model*

The CT26 tumor mouse model was established by subcutaneous injection of 1×10^6^ cells into the right hind thigh of female BALB/c mice. Tumor volume was calculated using the following formula:

Tumor Volume=Length of Tumor × Wid of Tumor2÷2*Tumor Volume=Length of Tumor × Wid of Tumor2÷2*

(10)

*1.4.3. Immunohistochemistry (IHC)*

Tumor tissues were collected from mice in each group, fixed, dehydrated, paraffin-embedded, and sectioned. Sections were then deparaffinized, subjected to antigen retrieval, blocked, and treated with primary and secondary fluorescent antibodies followed by DAPI staining. Finally, collected images were observed under a fluorescence microscope.

*1.4.4. Histological Analysis and Safety*

Following the completion of treatment, major organs (including heart, liver, spleen, lung, and kidney) were collected from mice in each group. These organs were fixed in 4% paraformaldehyde, dehydrated, embedded in paraffin, sectioned, and stained with hematoxylin and eosin (H&E) for histological analysis. Tumor tissues were collected from mice in each group, fixed, dehydrated, paraffin-embedded, and sectioned. Sections were stained with H&E and analyzed using a TUNEL apoptosis detection kit. Sections were observed on an Olympus BX-51 optical system for histological analysis.

1. **Supplementary Figurers**


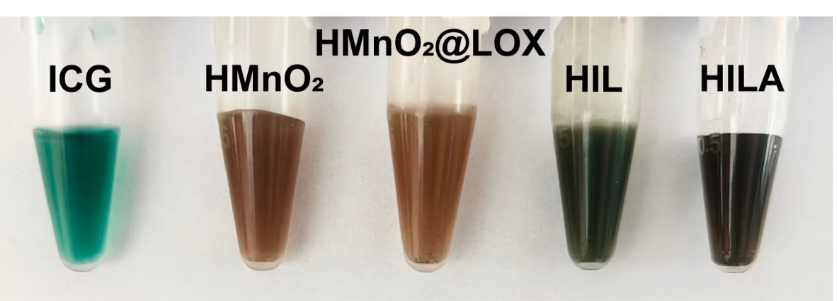


**Figure S1. Photographs of all intermediates and final construct during the functionalization process of HMnO_2_.**


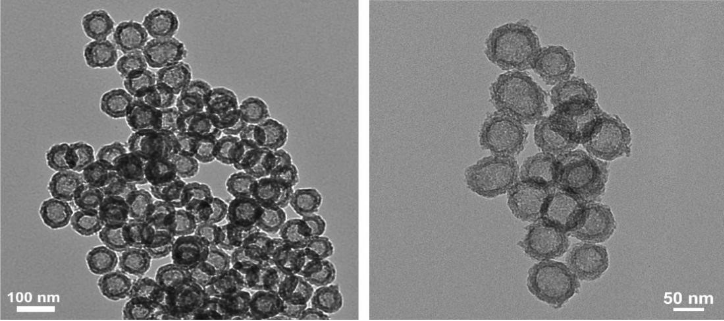


**Figure S2. TEM image of HMnO_2_.**


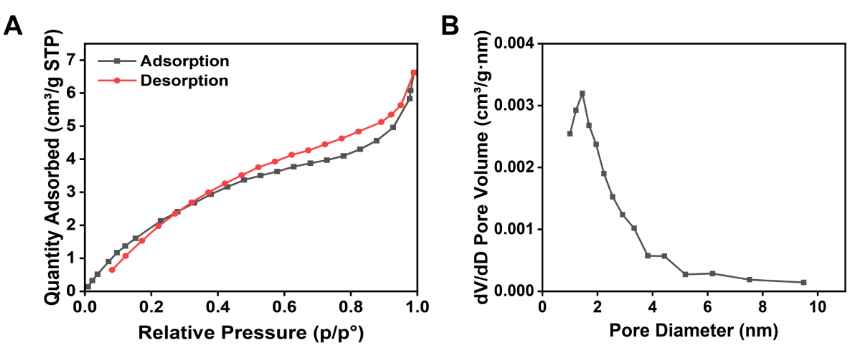


**Figure S3. N_2_ adsorption/desorption isotherms (A) and pore size distribution curve (B) for HMnO_2_.** The specific surface area was measured to be 10.15 m^2^/g, and the mesopore size distribution was predominantly centered within the 1.0–3.0 nm range, with a mean pore diameter of 2.50 nm.


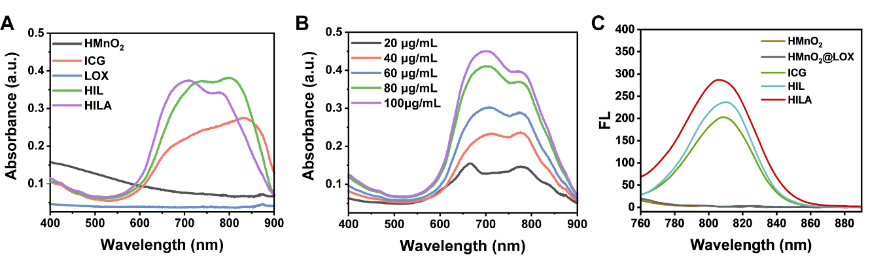


**Figure S4. UV-vis absorption spectra and fluorescence spectra of HILA and its individual components.** (A) UV-vis absorption spectra of HILA and its individual components. (B) UV-vis absorption spectra of HILA aqueous solutions at different concentrations. (C) Fluorescence spectra of HILA and its individual components.


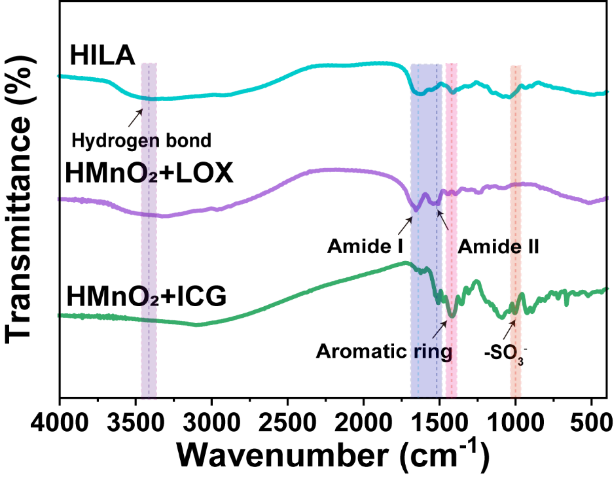


**Figure S5. FT-IR spectra of HILA and physical mixtures (ICG+HMnO_2_ and LOX+HMnO_2_).**


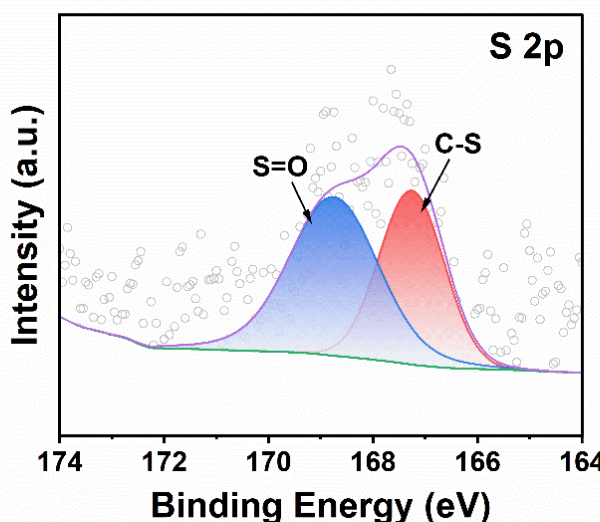


**Figure S6. High-resolution scans of S 2p peaks in HIL.**


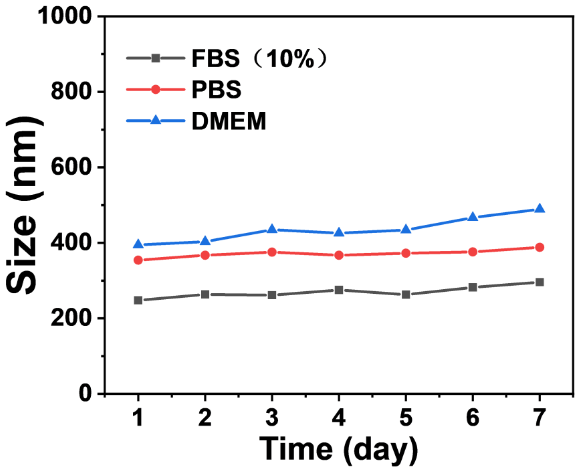


**Figure S7. Changes in hydrated particle size of HILA (100 μg/mL) in different physiological solutions (10% FBS, PBS, and DMEM).**


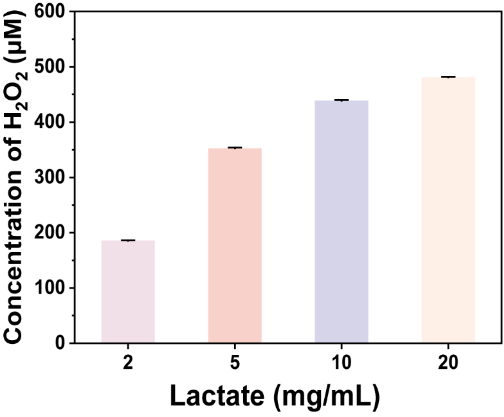


**Figure S8. H_2_O_2_ production upon reaction of HILA with lactate at various concentrations (2, 5, 10, 20 mg/mL).**


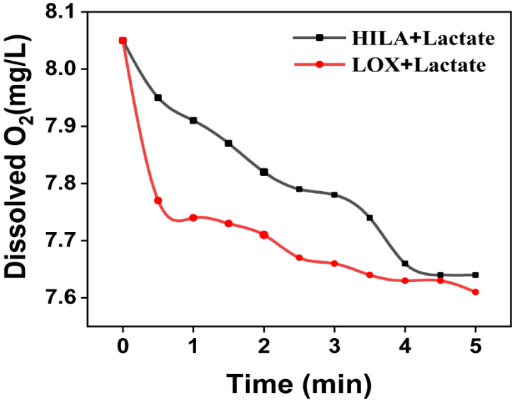


**Figure S9. Comparison of O_2_ consumption between HILA and free LOX during lactate catalysis.**


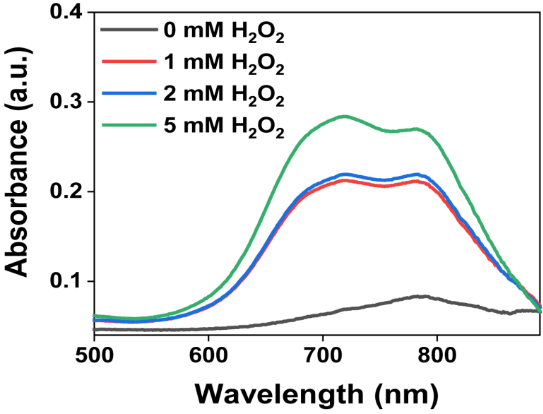


**Figure S10. UV absorption spectra of ICG release from HILA in solutions of varying concentrations of H_2_O_2_.**


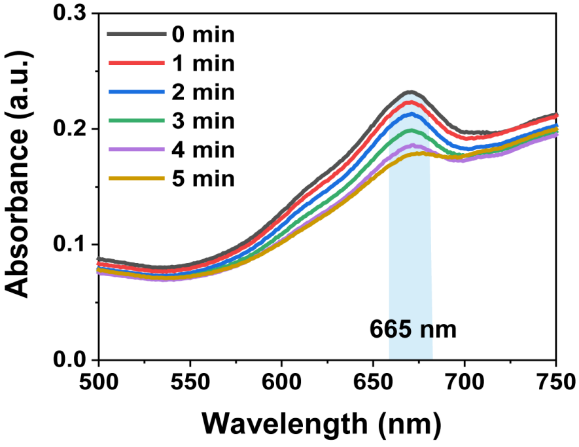


**Figure S11. Changes in MB absorption spectra upon HILA reaction with 10 mM H_2_O_2_ at different time points (0, 1, 2, 3, 4, and 5 min) under pH 5.0 and 10 mM GSH conditions.**


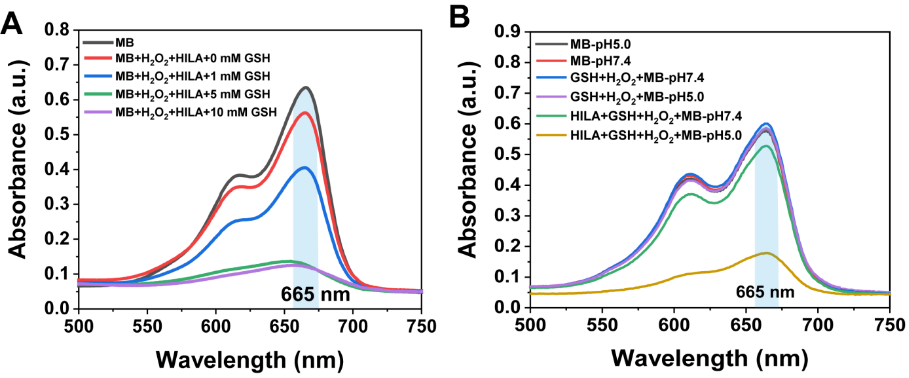


**Figure S12. The mechanism of •OH generation through the interaction between HILA and H_2_O_2_.** (A) Changes in MB absorption spectra after HILA (100 μg/mL) reacted with different GSH concentrations (0, 1, 5, and 10 mM). (B) Changes in the MB absorption spectrum after HILA (100 μg/mL) reaction under 5 mM GSH, 10 mM H_2_O_2_ and different pH conditions (5.0 and 7.4).


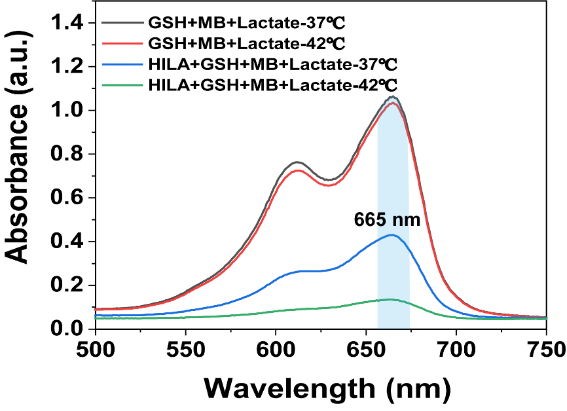


**Figure S13. Changes in MB absorption spectra of HILA under different temperatures (37°C and 42°C) in a lactate (40 mM) tumor tissue simulation environment.**


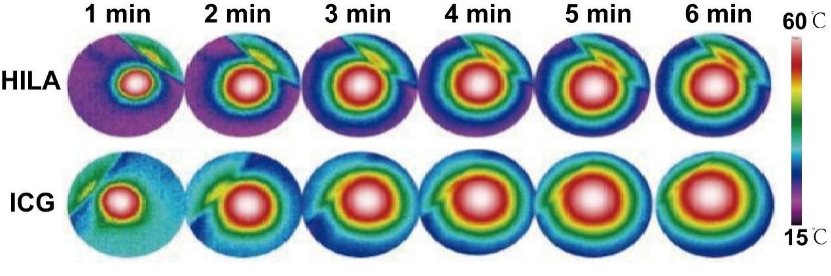


**Figure S14. Infrared thermal imaging photographs of ICG and HILA (150 μg) in a 96-well plate after laser irradiation.**


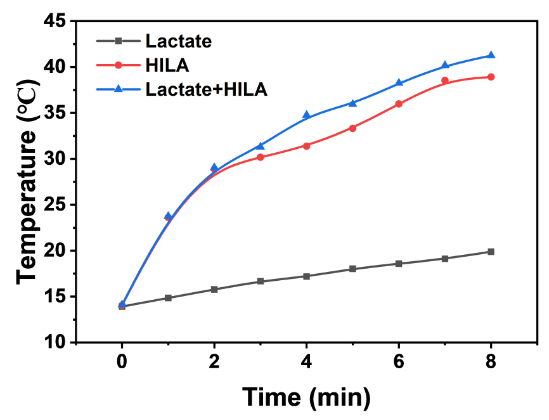


**Figure S15. Temperature rise curve of HILA under simulated tumor tissue lactate conditions (40mM).**


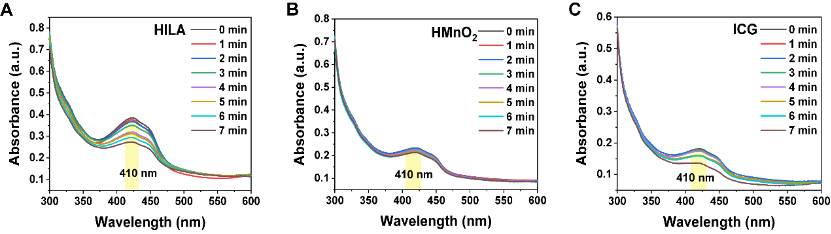


**Figure S16. (A–C) UV absorption spectra of DPBF probe at different time points during 7-min laser irradiation for HILA (A), HMnO_2_ (B), and ICG (C).**


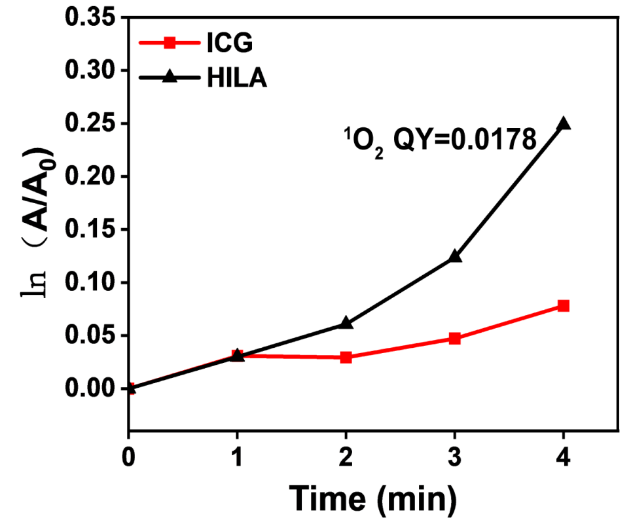


**Figure S17. The ROS quantum yield of HILA.**


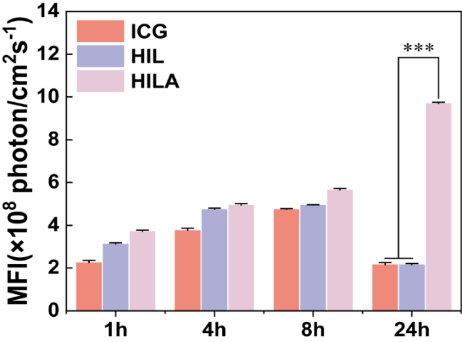


**Figure S18. Change in average fluorescence intensity of the tumor region during fluorescence imaging in mice.** *** *p* < 0.001.


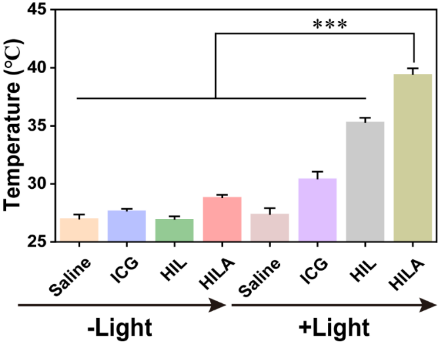


**Figure S19. Temperature changes after laser irradiation of tumor sites in different treatment groups.** *** *p* < 0.001.


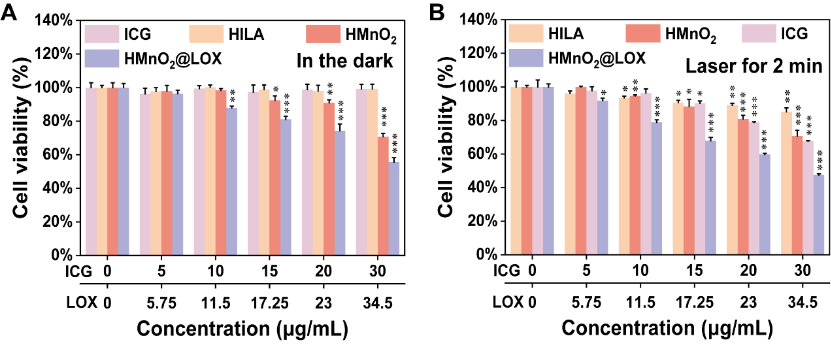


**Figure S20. *In vitro* safety evaluation of HILA.**  Cytotoxicity of different administration groups on L-929 cells under dark conditions (A) and under laser irradiation (B). Data are presented as mean ± SD (n=5). Statistic significances between every two groups were determined by *two-tailed Student’t-test*. * *p* < 0.05, ** *p* < 0.01, and *** *p* < 0.001 vs. the 0 μg/mL group.


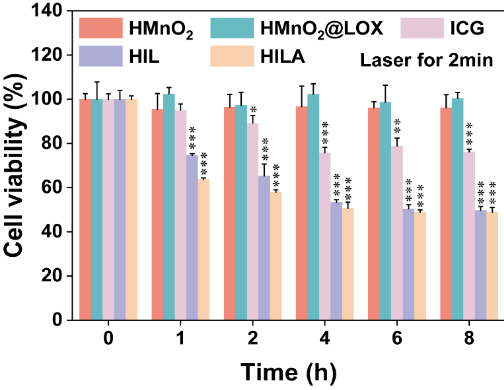


**Figure S21. Cytotoxic effects of each formulation group (ICG concentration: 10 μg/mL) on CT26 cells at different incubation times.** Data are presented as mean ± SD (n=5). Statistic significances between every two groups were determined by *two-tailed Student’s t-test*. * *p* < 0.05, ** *p* < 0.01, and *** *p* < 0.001 vs. the 0 μg/mL group.


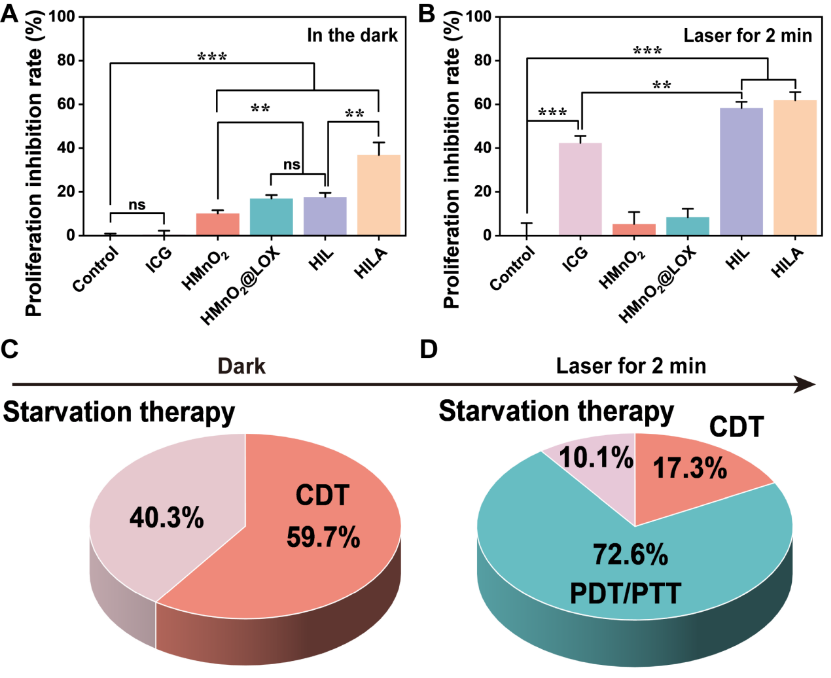


**Figure S22. Contributions of CDT, starvation therapy, and PDT/PTT to the overall antitumor efficacy of HILA.** (A) Proliferation inhibition rates of each formulation group (ICG: 20 μg/mL) under dark conditions. (B) Proliferation inhibition rates of each formulation group (ICG: 10 μg/mL) under laser irradiation. Contribution ratios of different treatment modalities under dark conditions (C) and laser irradiation (D). Data are presented as mean ± SD (n=5). *ns*, no significance, ** *p* < 0.01, and *** *p* < 0.001 vs. the control group.


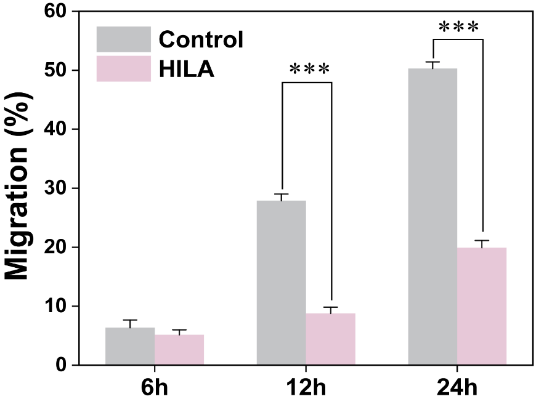


**Figure S23. Quantitative statistical analysis of cell migration rates.** *** *p* < 0.001.


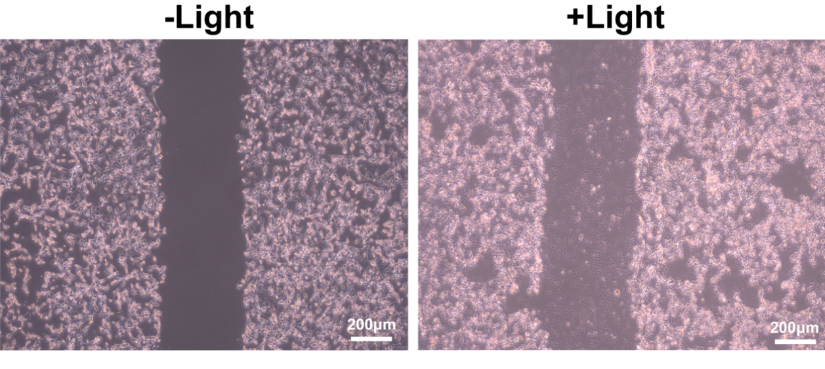


**Figure S24. Cell images from scratch assays in the HILA and HILA+L groups.**


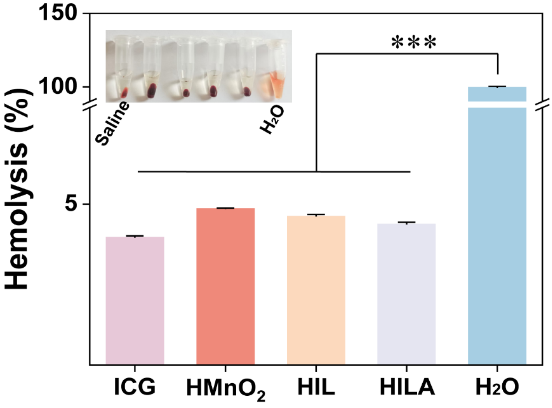


**Figure S25. Hemolysis rates after incubation of different nanoparticles with red blood cells (5%).** *** *p* < 0.001.


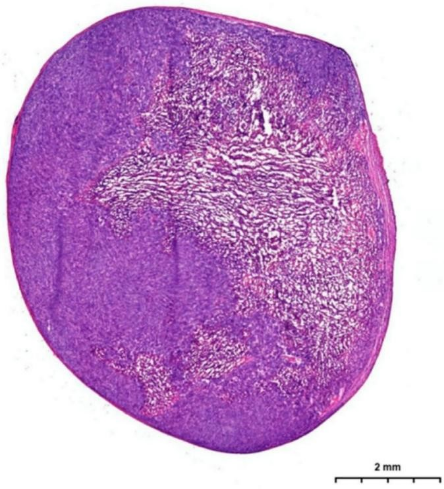


**Figure S26. Panoramic view of tumor HE staining in the HILA+L treatment group.**


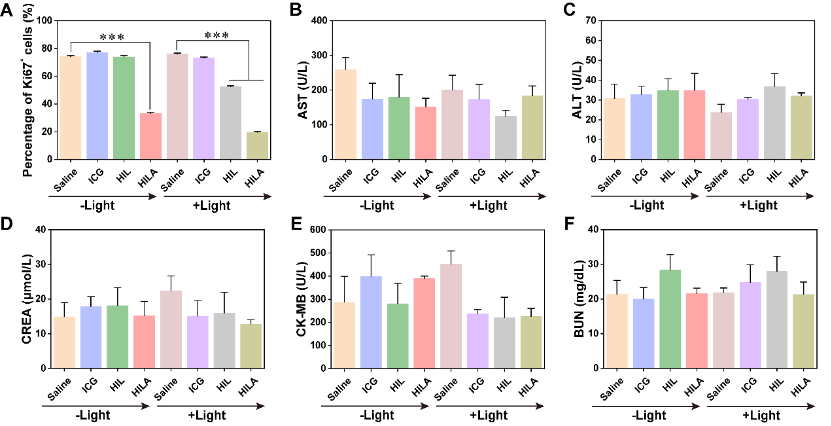


**Figure S27. Quantification of Ki67-positive rates and partial blood biochemical indicators of mice in different groups.** (A) Comparison of Ki67-positive tumor cell levels across different treatment groups. Biochemical indicators in mice from different treatment groups, including AST, ALT, CREA, CK-MB, and BUN (B-F). *** *p* < 0.001.


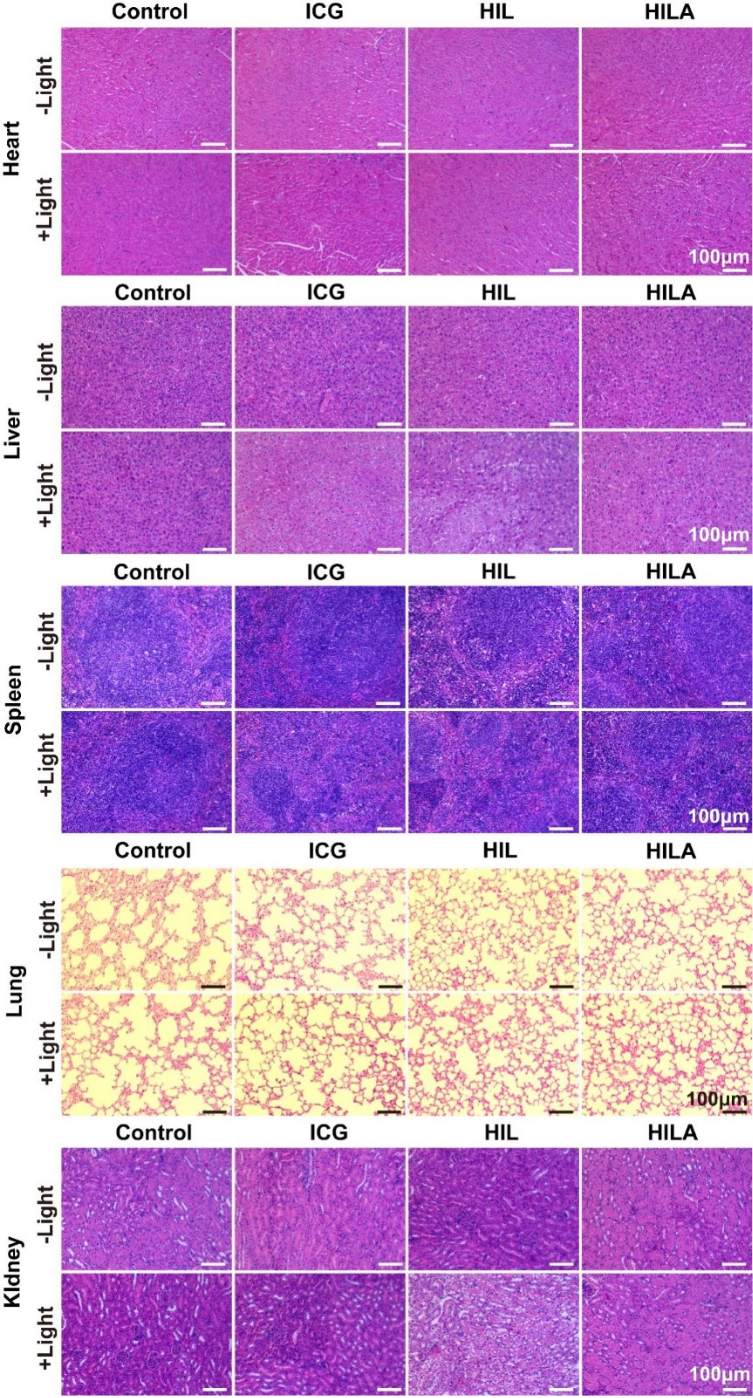


**Figure S28. H&E staining images of the main organ slices of mice from different groups (scale bar=100 μm).**

**3. Supplementary Tables**

Table S1. ICG loading and encapsulation rates in HILA (n=3)

| Batch | ICG Loading (%) | Encapsulation rates of ICG（%） | |
| --- | --- | --- | --- |
| 1 | 13.86 | | 82.63 |
| 2 | 14.31 | | 85.26 |
| 3 | 14.16 | | 84.40 |
| Average date | 14.11 ± 0.23 | | 84.10 ± 1.34 |

Table S2. LOX loading and encapsulation rates in HILA (n=3)

| Batch | LOX Loading (%) | Encapsulation rates of LOX（%） |
| --- | --- | --- |
| 1 | 15.51 | 77.05 |
| 2 | 17.37 | 86.25 |
| 3 | 16.32 | 81.05 |
| Average date | 16.40 ± 0.93 | 81.45 ± 4.61 |

Table S3. Comparison of HILA with reported materials

| Parameters | HILA | ICG (free) | Black Phosphorus (BP) | Gold Nanomaterial |
| --- | --- | --- | --- | --- |
| η (%) | 33.83 | < 20 (typical) | 22.6–28.4 (BPQDs/BPs) ^[1]^ / 43.6–64.2 (modified) ^[2]^ | Shape-dependent (~36% higher for star-shaped vs. spheres) ^[3]^ |
| Thermal stability | Moderate | Poor (severe photobleaching, 14.85 °C decrease) | Poor (rapid oxidation/degradation) ^[2]^ | Excellent |
| Biodegradability | Degradable (HMnO_2_→Mn^2+^; ICG→ excreted) | Fast clearance | Biodegradable (to PO_4_^3-^) ^[2]^ | Non-biodegradable, long-term retention ^[3]^ |
| FDA approval | Yes (ICG component) | Yes | No | No |
| Clinical translation potential | High | Moderate | Limited (stability concerns) | Moderate (toxicity concerns) |

Table S4. Pharmacokinetic parameters of each treatment group (n=3)

| Group | *t*_1/2α_(h) | *CL*(L/h/kg) | AUC_0-t_(mg/L*h) | MRT_0-t_(h) |
| --- | --- | --- | --- | --- |
| ICG | 0.31 ± 0.02 | 4468.94 ± 0.95 | 1.74 ± 0.03 | 2.87 ± 0.09 |
| HIL | 2.30 ± 0.09 | 1674.98 ± 0.55 | 5.50 ± 0.60 | 3.65 ± 0.28 |
| HILA | 6.49 ± 0.08 | 633.43 ± 0.09 | 15.28 ± 0.31 | 9.19 ± 0.15 |

**References**

1. X. Yang, D. Wang, Y. Shi, J. Zou, Q. Zhao, Q. Zhang, W. Huang, J. Shao, X. Xie and X. Dong, “Black Phosphorus Nanosheets Immobilizing Ce6 for Imaging-Guided Photothermal/Photodynamic Cancer Therapy,” *ACS Appl Mater Interfaces* (2018): 12431, <https://doi.org/10.1021/acsami.8b00276>.
2. Z. Li, H. Xu, J. Shao, C. Jiang, F. Zhang, J. Lin, H. Zhang, J. Li and P. Huang, “Polydopamine-Functionalized Black Phosphorus Quantum Dots for Cancer Theranostics,” *Applied Materials Today* (2019): 297, <https://doi.org/10.1016/j.apmt.2019.02.002>.
3. a) Z. Dong, X. Lu, W. Zhang, S. Tang, H. Liu, R. Zhang, Y. Liang, X. Liu and Y. Zhang, “Current Applications and Future Perspectives on Rare-Earth-Based Materials in Stomatology,” *iScience* (2025): 113220, <https://doi.org/10.1016/j.isci.2025.113220>; b) M. F. Amézaga González, J. Acosta Bezada, V. Gómez Flores, C. Chapa González, J. R. Farias Mancilla, S. J. Castillo, C. Avila Orta and P. E. García-Casillas, “Effect of Physiological Fluid on the Photothermal Properties of Gold Nanostructured,” *Int J Mol Sci* (2023), <https://doi.org/10.3390/ijms24098339>; c) M. S. Rashwan, M. M. Alam, S. H. Jaberi, A.-A. Al-Sheikh, A. C. S. Samia, H. Baskaran and C. Burda, “Plasmonic Enhancement of Photothermal Conversion in Hydrogels using Gold Nanorods,” *Materials Advances* (2025): 5558, <https://doi.org/10.1039/d5ma00278h>.
